# Efficient method for isolation of reticulocyte RNA from healthy individuals and hemolytic anaemia patients

**DOI:** 10.1111/jcmm.13951

**Published:** 2018-11-18

**Authors:** Michał Skulski, Rafał Bartoszewski, Michał Majkowski, Beata Machnicka, Kazimierz Kuliczkowski, Aleksander F. Sikorski, Dżamila M. Bogusławska

**Affiliations:** ^1^ Department of Cytobiochemistry Faculty of Biotechnology University of Wrocław Wrocław Poland; ^2^ Department of Biology and Pharmaceutical Botany Faculty of Pharmacy with Subfaculty of Laboratory Medicine Medical University of Gdańsk Gdańsk Poland; ^3^ Department of Biochemistry and Bioinformatics Faculty of Biological Sciences University of Zielona Góra Zielona Góra Poland; ^4^ Department of Haematology, Blood Neoplasms and Bone Marrow Transplantation Wroclaw Medical University Wrocław Poland; ^5^ Present address: Department of Tumor Immunology Polish Academy of Sciences Laboratory of Molecular and Cellular Immunology Hirszfeld Institute of Immunology and Experimental Therapy Wrocław Poland

**Keywords:** hereditary hemolytic anaemia, hereditary spherocytosis, reticulocyte isolation, reticulocyte transcriptome, RNA‐Seq

## Abstract

Despite enormous progress and development of high‐throughput methods in genome‐wide mRNA analyses, data on the erythroid transcriptome are still limited, even though they could be useful in medical diagnostics and personalized therapy as well as in research on normal and pathological erythroid maturation. Although obtaining normal and pathological reticulocyte transcriptome profiles should contribute greatly to our understanding of the molecular bases of terminal erythroid differentiation as well as the mechanisms of the hematological diseases, a basic limitation of these studies is the difficulty of efficient reticulocyte RNA isolation from human peripheral blood. The restricted number of possible parallel experiments primarily concern healthy individuals with the lowest number of reticulocytes in the peripheral blood and a low RNA content. In the present study, an efficient method for reticulocyte RNA isolation from healthy individuals and hemolytic anaemia patients is presented. The procedure includes leukofiltration, Ficoll‐Paque gradient centrifugation, Percoll gradient centrifugation, and negative (CD45 and CD61) immunomagnetic separation. This relatively fast and simple four‐stage method was successfully applied to obtain a reticulocyte‐rich population from healthy subjects, which was used to efficiently isolate the high‐quality RNA essential for successful NGS‐based transcriptome analysis.

## 1 INTRODUCTION

Reticulocytes are non‐nucleated immature red blood cells in peripheral blood of mammalian species. Young cells contain residual RNA and micro‐organelles such as ribosomes, lysosomes, and mitochondria. The residual RNA is not only functional, but also necessary for the final stages of reticulocyte maturation.^1^, ^2^ Transformation from a reticulocyte to a fully mature red cell is a complex process requiring substantial remodelling of the erythrocytic cytoplasm and membrane.^3^ Maturation of the reticulocyte is a 2‐3‐day‐long process, during which hemoglobin synthesis occurs, and during the last days old reticulocytes eliminate their residual RNA and are released into the circulation, to become mature red blood cells.^4^ The presence of an intracellular network of residual reticulocyte RNA allows one to differentiate between various stages of the development of reticulocytes. The detection of reticulocyte RNA with brilliant cresyl blue allows one to distinguish reticulocytes from mature red blood cells as well as enabling the identification of the youngest, highly fluorescent reticulocytes (HFR), supplied early from bone marrow in conditions of increased erythropoietic stimulation, eg, hemolytic anaemia. An example of such conditions is increased hemolysis due to red blood cell pathology. Importantly, in hemolytic anaemia both HFR and the reticulocyte count are markedly increased and inversely correlated with hemoglobin (Hb) level.^5^ Hence, reticulocytes (as well as their transcriptome) may be useful in the diagnostics of hematopoietic system diseases including hemolytic anaemia.

Adult peripheral blood is characterized by a low reticulocyte count resulting in an extremely low yield of reticulocyte RNA isolation as compared to cord blood.^6^ Therefore, few parallel experiments on healthy individuals’ reticulocytes are possible due to having the lowest number of reticulocytes in their peripheral blood. Furthermore, although reticulocyte transcriptome analyses could greatly improve our understanding of reticulocytes’ maturation, they require relatively high quality of reticulocyte RNA free of RNA derived from other blood cells. Only few procedures for peripheral blood reticulocyte isolation are available in the literature.^7^, ^8^ Taken together, developing novel efficient methods for quality reticulocyte RNA isolation constitutes an essential step in further studies on their maturation.

Herein we propose a novel, simple, and effective method for the isolation of reticulocyte RNA from both healthy individuals and hemolytic anaemia patients. The method was validated on blood samples obtained from Polish individuals who were adult males (aged 25‐40 years): (a) three healthy individuals (including one blood donor), (b) one hereditary spherocytosis (HS) patient^9^, ^10^, ^11^ and (c) two hereditary nonspherocytic hemolytic anaemia (HA) patients (brothers). The four‐step reticulocyte isolation procedure presented here can be performed within a few hours of blood donation, as recommended by RNA isolation procedures. Finally, the proposed method allows efficient and specific isolation of reticulocyte RNA, which can be successfully used for detection of specific transcripts by both qPCR and RNA‐Seq (RNA sequencing).

## 2 MATERIAL AND METHODS

### 2.1 Healthy individuals and patients

EDTA anticoagulated blood was collected from volunteers (healthy individuals, C1, C2, including one blood donor, C3), HA patients and HS patient by venipuncture (Supporting Information Table S1). During our studies on hereditary spherocytosis, we identified a family in which two members (brothers, N61, N62) displayed symptoms of hemolytic anaemia that did not fit the characteristics of the known disease. Both patients revealed the presence of stomatocytes (as well as anisocytosis and azurophilic stipplings) but were depleted of spherocytes typical of hereditary spherocytosis in their blood smears.

A HS patient with moderate symptoms of the disease was recruited (patient C14, not splenectomized, *SPTB* mutation coming from two‐generation Polish C family).^11^ The Ethics Committee of Wroclaw Medical University approved the study protocol KB‐541/2011. Informed consent was obtained from all individuals before entering the protocol.

### 2.2 Flow cytometry phenotyping

Flow cytometric analysis was performed using a BD FACSCalibur flow cytometer to ensure the purity of the isolated CD71^+^ cells. Packed reticulocytes were directly stained with anti‐human CD71‐PE antibody (Miltenyi Biotec, Bergisch‐Gladbach, Germany) to identify the different reticulocyte populations relative to RNA content as measured by Thiazole Orange (TO). Thiazole Orange (Sigma‐Aldrich, St. Louis, MO, USA) was dissolved in methanol to obtain a 1 mg/mL TO stock solution. The stock solution was kept at −20°C in darkness.

A TO working solution (20 μg/mL) was prepared by adding 6 μL of stock solution to 30 mL of PBS (pH 7.4).

Samples were stained as follow: 1.3 μL of peripheral blood (or 3 μL of isolated reticulocytes sample) was added to 2.5 mL of TO working solution containing 6 μL of anti‐CD71‐PE antibody (Miltenyi Biotec) or 6 μL of an isotype control (Mouse IgG2a‐PE; Miltenyi Biotec). After 20 minutes incubation samples were analysed. The FL‐1 channel (BP 530/30) was employed to detect TO fluorescence whereas the FL‐2 channel (BP 585/42) was employed for PE fluorescence. Single stained samples were used for setting the correct fluorescence compensation.

### 2.3 Purification of reticulocytes from human peripheral blood

**Figure 1:**
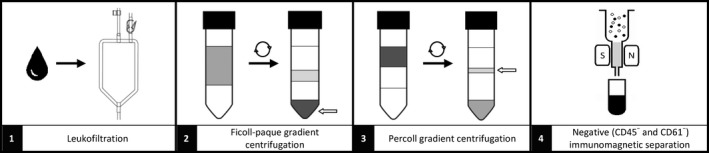
Scheme of four‐step purification procedure used to obtain reticulocyte‐rich suspension from human peripheral blood

The procedure of purification of reticulocytes from human peripheral blood included four steps (Figure 1):

1. Fresh blood from volunteers and patients immediately after drawing was diluted with PBS‐EDTA pH 7.4 buffer (27 mL: 70 mL) and was passed through a LEUCOBED LCG3 filter (Macopharma, London, UK) to remove most leukocytes and platelets.
2. The obtained filtrate (approximately 50 mL) was divided into two equal parts, each placed onto separate Ficoll‐Paque PREMIUM density gradient (1.077 g/mL, GE Healthcare Bio‐Science, Uppsala, Sweden) and centrifuged with reduced acceleration and deceleration (40 minutes, 400 g at 4°C). Then cells were washed with PBS‐EDTA pH 7.4 buffer (8 minutes, 600 g at 4°C). The supernatants were removed gently, leaving 5 mL above the cell pellets. Finally, each of the cell pellets was resuspended in the remaining supernatant volume.
3. The selective removal of red blood cells was performed using a Percoll density gradient (GE Healthcare Bio‐Science). Half the volume of each cell suspension obtained above (2.5 mL) was applied on one of four cold gradients (*d* = 1.096 g/mL and 1.058 g/mL in Falcon, 15 mL test tube) and centrifuged for 30 minutes (250 g at 4°C). Each reticulocyte‐rich layer was subsequently collected into 50 mL centrifuge tubes and washed twice in PBS‐EDTA pH 7.4 buffer (8 min, 550 g at 4°C) to obtain 1 mL of reticulocyte‐rich suspension, 50% of which were reticulocytes and ˜50% red blood cells. Nevertheless, a few leukocytes and platelets were still present. Then reticulocyte‐rich suspensions were transferred into 2 mL test tubes and washed again under the same conditions. Finally, each of the cell pellets was resuspended in the remaining supernatant (˜2 mL) and centrifuged (8 minutes, 550 g at 4°C). Supernatants were gently removed, and the number of reticulocytes and red blood cells determined.
4. Finally, to remove remaining leukocytes and platelets from the reticulocyte‐rich suspensions, negative immunomagnetic selection (CD45 MicroBeads and CD61 MicroBeads; Miltenyi Biotec) using the MiniMACS Separator System was applied, in accordance with the manufacturer's procedure with a slight modification. Namely, each reticulocyte‐rich pellet obtained as described above was resuspended in 80 μL of PBS‐EDTA‐BSA pH 7.4 buffer and into each cell suspension 20 μL of CD45 MicroBeads as well as 5 μL of CD61 MicroBeads simultaneously was added. After an additional 15 minutes incubation, the separation was carried out at the appropriate time intervals (the time of a single separation is about 10 minutes) using four MS columns and one MACS Separator (Miltenyi Biotec). The combined flow‐through containing reticulocytes/RBC was centrifuged (10 minutes, 300 g at 4°C) to obtain the final reticulocyte‐rich pellet which still contained about 50% RBC. The supernatant was removed to leave 0.4 mL above the cell pellets in which the cells were resuspended. To assess the purity 20 μL of reticulocyte‐rich pellet was directly mixed with 1% Brilliant Cresyl Blue (w/v in 0.9% sodium chloride) in the ratio 1:1, incubated for 30 minutes (RT) and visualized using an inverted microscope with differential interference contrast (DIC)—see Supporting Information Figure S1.

### 2.4 RNA isolation and cDNA synthesis

Total RNA was extracted from the reticulocyte‐rich suspension and collected using the miRNeasy Mini Kit (Qiagen, Hilden, Germany), according to the manufacturer's recommendation. In the first isolation step, ~0.4 mL of the reticulocyte‐rich pellet with the supernatant was divided into two aliquots. In further steps we followed the manufacturer's procedure until the material was applied onto an RNeasy Mini Spin Column. During this step, the materials from two aliquots were pooled (to enhance yields of RNA isolation), to ensure fulfilling the minimal column capacity required for efficient RNA isolation. RNA concentrations were calculated based on the absorbance at 260 nm. RNA samples were stored at −70°C. The first strand of cDNA was synthesized using a RevertAid First Strand cDNA Synthesis Kit (Thermo Fisher Scientific, Waltham, MA, USA).

### 2.5 Assessment of purity of isolated reticulocyte population by RT‐PCR

To verify the purity of the reticulocyte population the RNA isolates from reticulocyte populations was tested using specific primers for marker gene transcripts: *HBB*,* PPARA*,* PTPRC*,* ITGB3*, and *ACTB*. Primer sequences are listed in Supporting Information Table S2. A two‐step RT‐PCR was applied (DreamTaq DNA Polymerase; Thermo Fisher Scientific).

### 2.6 Next generation RNA sequencing analyses

Next generation sequencing was performed by Heflin Center for Genomic Science Core Laboratories, University of Alabama at Birmingham, AL, USA. Directly prior to the RNA‐Seq procedure, the total RNA integrity was verified by on‐chip electrophoresis (Bioanalyzer, Agilent Technologies, Santa Clara, CA, USA) and only samples with high RNA integrity (RIN) values were further processed (>7.5). In addition, quality score analysis was performed confirming the high quality of RNA samples. Following rRNA depletion, the remaining RNA fraction was used for library construction and subjected to 100 bp paired‐end sequencing on an Illumina HiSeq 2000 instrument. Sequencing reads were aligned to the human reference genome assembly (hg19) using TopHat.^12^ Transcript assembly and estimation of the relative abundances were carried out with Cufflinks.^13^

### 2.7 Bioinformatic analysis of transcriptome biological context

GeneAnalytics (geneanalytics.genecards.org) is a comprehensive gene set analysis tool for contextualization of expression patterns and functional signatures obtained from the post‐genomics Big Data sets, such as next generation sequencing (NGS), RNA‐Seq, and microarray experiments.^14^ The webserver was used to place RNA‐Seq results into a physiological context using a built‐in biological pathway algorithm. In GeneAnalytics, matched SuperPaths appear with their matching score and link to the relevant webcard in PathCards, as well as the list of matched genes and total number of genes associated with each SuperPath. The scoring algorithm in the pathways category is based on the algorithm used by the GeneDecks Set Distiller tool.^15^ Briefly, all genes in each SuperPath are given a similar weight in the analysis, and the matching score is based on the cumulative binomial distribution, which is used to test the null hypothesis that the queried genes are not over‐represented within any SuperPath pathway. Unification is employed on all of the sources found in GeneCards. When using GeneAnalytics we focus our analysis only on expression signatures that were assigned with high scores, which reflects high biological pathway/phenotype coverage. The gene nomenclature was adjusted to the webserver requirements (GeneCards).

## 3 RESULTS

### 3.1 Reticulocyte isolation

**Figure 2:**
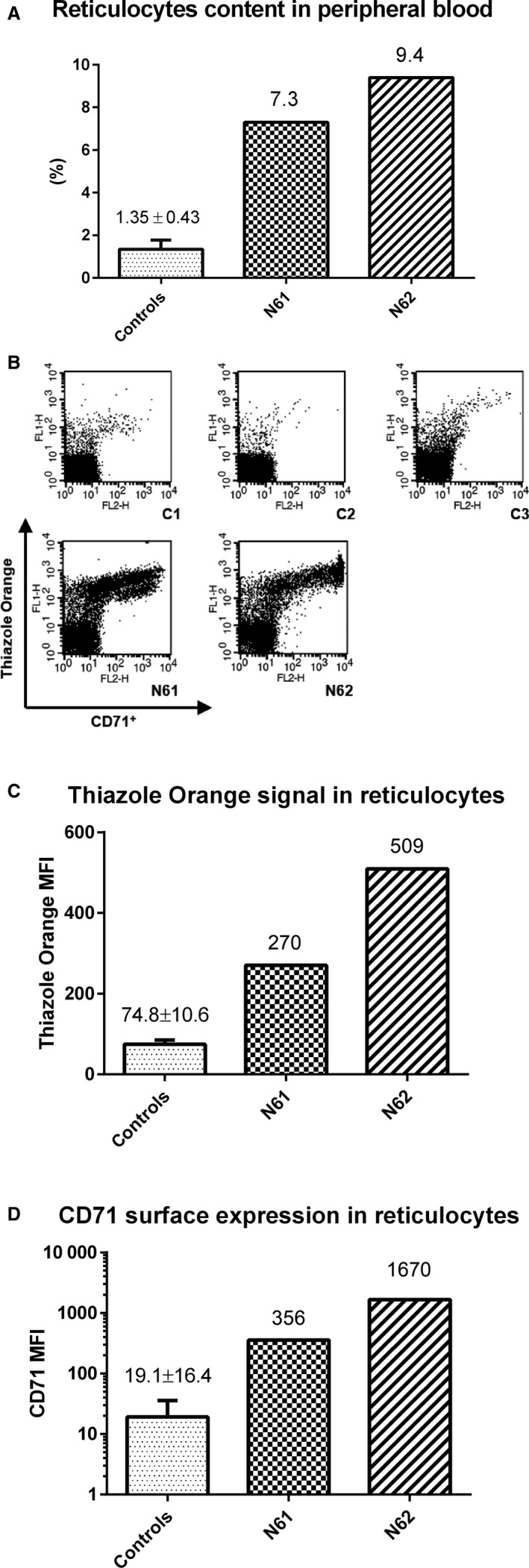
Reticulocyte characteristics. A, Examples of reticulocyte quantification in human peripheral blood. Examination was performed for healthy individuals (C1‐C3) and for hemolytic anaemia patients (N61 & N62). The number of reticulocytes was determined from the dot plots: FSC (forward scatter) vs TO fluorescence. B, Analysis of CD71 surface exposure in WBC and platelet‐free reticulocyte preparations obtained from the healthy individuals and HA patients allows determination of reticulocyte number and maturity status. Dot plots showing CD71 surface expression and RNA content (TO) in reticulocytes isolated from healthy individuals and HA patients (N61, N62). These data indicate a significantly higher number of immature (early) reticulocytes in peripheral blood of HA patients than in healthy individuals. C, Quantification of purified reticulocyte measured by Thiazole Orange (TO, RNA content) fluorescence reveals 3.6 and 6.7 times higher signals for N61 and N62 HA patients, respectively, than in healthy individuals (controls: n = 3). D, CD71 surface expression in purified reticulocytes for N61 and N62 HA patients is significant

Fresh peripheral blood was collected from healthy volunteers, patients with hereditary spherocytosis and hemolytic anaemia. The mean age of the volunteers/patients (6 men) was 34 years (25‐40). Differences in the reticulocyte count in hemolytic anaemia patients and healthy individuals are shown in Figure 2A. To obtain a reticulocyte‐rich population free of nucleated blood cells we applied a four‐step procedure (Figure 1, Section 2). In the first step, the whole blood was filtered through a leukocyte removal filter. Then the resulting blood filtrate was centrifuged on the Ficoll‐Paque Premium density gradient followed by further purification of reticulocytes by Percoll density gradient centrifugation.^16^ The former step was repeated once to remove most of the red blood cells, as their presence prevented correct execution of the next steps. The above‐mentioned steps of the presented procedure were still not sufficient to obtain a pure reticulocyte population. Monocytes and platelets (which also contained RNA) were still present in our microscopic preparations. Hence, to remove the residual nucleated cells and platelets the MACS magnetic cell separation technology was applied. Initially, a method of positive selection of reticulocytes with the CD71 MicroBeads was used. However, this approach was successful for patients with high reticulocytosis only. In healthy subjects, CD71^+^ cells were so scarce that no effective RNA isolation was possible. For this reason, we decided to use negative selection of leukocytes and platelets using CD61 and CD45 MicroBeads. The resulting reticulocyte preparation, although containing a reasonable number of red blood cells (Supporting Information Figure S1), provided a starting material for efficient RNA isolation in which a highly purified reticulocyte population is needed. An effective method to differentiate various stages of the development of reticulocytes is the use of Thiazole Orange (TO) staining,^17^ which can be substituted for another commonly used reticulocyte classification system known as the reticulocyte maturity index (RMI).^18^ The most effective method of determining the cord reticulocyte subset with minimal artifacts is the use of anti‐CD71 antibodies and magnetic separation.^6^ The CD71 positive group characterizes the early human reticulocytes (Heilmeyer groups I to III) and the mature CD71 negative subset (Heilmeyer group IV) which were found in normal circulation.^19^ To analyse the maturity status and the number of reticulocytes, the sample was incubated with Thiazole Orange (TO) and CD71‐PE markers and counted by flow cytometry (Figure 2B‐D). Results for each HA patient (N61 and N62) were compared independently to both controls (C1 and C14).

### 3.2 RNA isolation and cDNA synthesis

**Figure 3:**
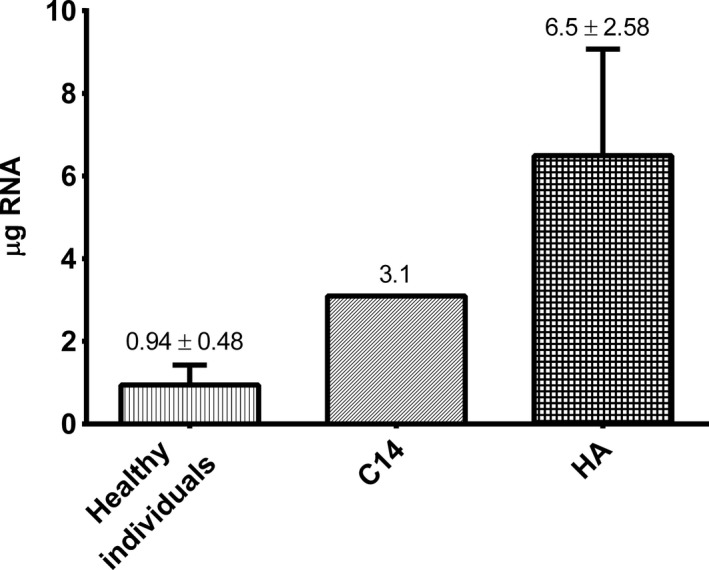
RNA amounts for each examined group. Healthy individuals (n = 4), C14—hereditary spherocytosis patient and HA—hemolytic anaemia patients (n = 2)

In order to test the usefulness of the described procedure of reticulocyte purification the functional quality of RNA was tested. RNA isolation was carried out as described in the Section 2. The obtained RNA quantities were sufficient for RNA‐Seq analysis for all study groups (Figure 3). The amount of RNA obtained from patient C14 diagnosed with HS was relatively low despite the increased reticulocytosis, which in our opinion is a consequence of decreased osmotic resistance of the red blood cells/reticulocytes resulting in excessive hemolysis observed during the reticulocyte isolation procedure.

### 3.3 Assessment of purity of isolated reticulocyte population by RT‐PCR

**Figure 4:**
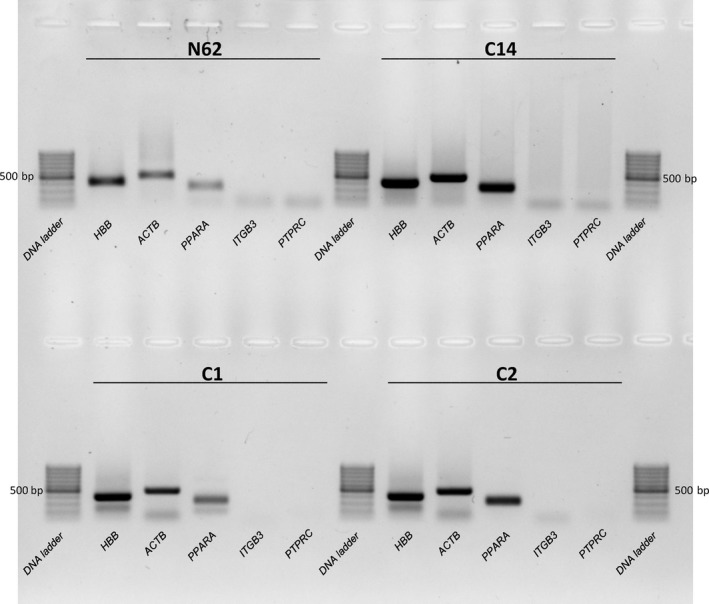
Example of reticulocyte cDNA quality control. Agarose gel electrophoresis of RT‐PCR products (image inverted, black/white) obtained using reticulocyte cDNA from patients (HA patient, N62, and HS patient, C14) and controls (healthy individuals: C1 and C2) and primers encoding sequences of the following genes: CD45 (*PTPRC* gene; 217 bp)—leukocyte marker; β‐globin (*HBB* gene; 397 bp)—erythroid marker; integrin‐β3 (*ITGB3* gene; 198 bp)—platelet marker. As the “low abundance” gene transcript *PPARA* (peroxisome proliferator activated receptor alpha; 324 bp) was chosen and was taken from the end of the reticulocyte cDNA library (NCBI, Library 11923). For the loading control β‐actin primers (*ACTB* gene; 479 bp) were used. All primer sequences used in this experiment are included in Supporting Information Table S2. As a standard “GeneRuler 100 bp DNA Ladder” (Thermo Fisher Scientific, Waltham, MA, USA) was used. Results show no leukocyte or thrombocyte RNA contamination for all analysed cases. The lowest band corresponds to primers

The reliability of RNA‐Seq analysis is based on obtaining a reticulocyte population free of contamination with other blood cells. Hence, to assess the reticulocyte purity we tested the obtained RNA isolates for the presence of high‐ and low‐abundant transcripts characteristic for these blood cells as well as for the absence of transcripts specific for platelets and leukocytes. As shown in Figure 4 and Supporting Information Figure S2, the RNA obtained from the isolated reticulocyte population contained only the gene transcripts that were reticulocyte‐specific: “high abundance” *HBB*, and “low abundance” *PPARA* (peroxisome proliferator‐activated receptor alpha, by an example which was taken from the end of the reticulocyte cDNA library; NCBI, Library 11923). As a quantitative control β‐actin (*ACTB*) primers were used. Furthermore, to verify efficient removal of leukocytes and platelets the RNA isolates from reticulocyte populations were tested. Transcripts specific markers for platelets (integrin β3 (*ITGB3*)) and for leukocytes (CD45 (*PTPRC*)) were absent.

### 3.4 Next generation RNA sequencing analyses—Expression status for erythroid and non‐erythroid transcripts

Reticulocyte transcriptome analysis using high‐efficiency gene expression assays was performed by RNA‐Seq for four male subjects only: both HA patients (N61 and N62), one healthy individual (C1) and one patient with HS diagnosis (C14, whose level of reticulocytosis was similar to that observed in the HA patients and the molecular basis of HS was known^11^). The use of two types of controls was due to the fact that only the most mature group IV reticulocytes, indicated by reticulocyte Heilmeyer^20^ maturation indices, are present in the peripheral blood of healthy individuals, which are rapidly transformed into red blood cells. Increased reticulocytosis seen in patients with hereditary hemolytic anaemia (including HS) usually results in a relatively high number of reticulocytes from groups II and III in peripheral blood.

Our reticulocyte transcriptome pattern was generated using the RNA‐Seq method and *Cufflinks* tool, the most popular RNA‐Seq analysis tool that assembles transcripts, estimates their abundances, and tests for differential expression and regulation in RNA‐Seq samples.^13^ Our analysis resulted in successful identification (positive reads status—number of reads allowing reliable location of its position in the nucleotide sequence) of 2685–2961 and 2465–2729 unique gene transcripts in healthy subjects and HS patient respectively. Importantly, among the identified gene transcripts the erythroid mRNAs were well represented, whereas non‐erythroid transcripts were absent (Table 1). These results indicate that the isolated population of reticulocytes is characterized by high homogeneity.

**Table 1:** Presence of selected erythroid and non‐erythroid transcripts obtained from RNA‐Seq data. Expression of genes was verified for both patients in the family N in comparison to two types of control: healthy subject (C1) and patient with HS diagnosis (C14)

Gene name	Gene expression			
	N61	N62	C1	C14
Erythroid genes: *ACTB, ADD1, ANK1, DMTN, EPB41, EPB42, GYPA, GYPB, GYPC,GYPE, MPP1, SLC4A1, SPTA1, SPTB, STOM*	+	+	+	+
Non‐erythroid genes: *ANK2, ANK3, ITGAL, ITGB3, PTPRC, SPTAN1, SPTBN1, SPTBN2, SPTBN4, SPTB5*	−	−	−	−

Using the GeneAnalytics web server, the results of RNA‐Seq analysis were convincingly (high score) assigned to 111 metabolic pathways, 104 biological processes, 31 molecular function pathways, 75 diseases, and 22 phenotypes (Supporting Information Table S3). Importantly, our set of gene transcripts was associated with 22 phenotypes that correlate with erythroid cells.

In conclusion, the method we developed for the isolation of a highly purified reticulocyte population from human peripheral blood from both healthy individuals and hemolytic anaemia patients is simple and effective. The four‐step isolation procedure can be performed within a few hours, as required by the RNA isolation procedures, while the quality and quantity of obtained RNA are sufficient for carrying out specific analysis of the transcriptome of reticulocytes.

## 4 DISCUSSION

Although reticulocytes constitute about 1% of the cell population in the human peripheral blood, the reticulocyte transcriptome remains poorly described. The RNA‐focused research on these cells is limited for several reasons: reticulocyte RNA isolation cannot be carried out from whole blood and excludes the use of methods of stabilization of intracellular RNA, reticulocytes have a low RNA content and contamination with nucleated cells may significantly affect the observed gene expression profile, and, most importantly, purification of reticulocytes from the peripheral blood of healthy individuals with normal reticulocyte levels and patients with hemolytic anaemia associated with increased osmotic fragility is usually of poor efficiency. An efficient purification procedure should fulfill the following criteria:

1. Isolation of RNA from pure reticulocytes excludes the use of modern methods of stabilization of intracellular RNA such as the PAXgene Blood RNA System. This is due to the fact that the PAXgene Blood RNA Tubes should be incubated for at least 2 hours at room temperature after blood collection to ensure complete lysis of all blood cells and thus subsequent isolation of selected blood cells is not possible. Therefore, the isolation procedure must be carried out quickly (it should take no longer than 5 hours) preferably from freshly drawn blood, after which RNA isolation should proceed. The procedure presented here meets this criterion.
2. The next issue is that cells such as red blood cells, reticulocytes, and platelets have a low RNA content.^21^, ^22^ Contamination of these cells with nucleated cells (containing a large amount of RNA) may significantly affect the observed gene expression profile. Based on our experience, use of a reticulocyte‐based purification procedure that relies only on leukofiltration and density gradient centrifugation yields only about 95% purity of the reticulocyte population, which essentially excludes further use of these preparations in transcriptomic analysis. Our procedure solves this problem by application of negative immunomagnetic selection with the MiniMACS Separator System and CD45 MicroBeads/CD61 MicroBeads.
3. Finally, variability in the reticulocyte population between healthy individuals and patients with hematologic diseases should be considered. Purification of reticulocytes from patients with hemolytic anaemia without changed osmotic resistance of red blood cells is relatively simple due to the increased reticulocyte count and no excessive hemolysis during isolation. Patients with hemolytic anaemia associated with increased osmotic fragility including conditions such as hereditary spherocytosis or hereditary elliptocytosis are characterized by high reticulocytosis, but usually during the isolation procedure increased hemolysis occurs, which significantly reduces the efficiency of isolation. In our procedure, we tried to optimize the method, to minimize hemolysis. However, the most challenging aspect is achieving efficient separation of the reticulocytes from the peripheral blood of individuals with a normal reticulocyte count, which has been pointed out in some reports.^6^ As mentioned above, in the peripheral blood of healthy individuals only the mature subset of CD71 negative reticulocytes is detected, whereas in patients with hemolytic anaemia, the early subset of CD71 positive reticulocytes is found. Therefore, when positive immunomagnetic selection with the MiniMACS Separator System and CD71 MicroBeads were used for healthy subjects, the obtained cell suspension contained a small number of reticulocytes and efficient RNA purification was not feasible. Furthermore, the RBC‐rich eluate was still contaminated with leukocytes and platelets. In the presented procedure application of negative immunomagnetic selection with the MiniMACS Separator System and CD45 and CD61 MicroBeads resulted in obtaining the flow‐through, containing only reticulocytes and red blood cells (1:1). The resulting amounts and purity of reticulocytes were sufficient for the efficient, high quality RNA isolation required in the NGS‐based transcriptome analyses (Table 1 and Figure 3). It should be noted that in the case of reticulocytes obtained from healthy individuals (where extensive hemolysis of red blood cells is not observed), the addition of globin reduction step may be considered, which can enrich data obtained from next generation sequencing‐based expression profiling.^23^

**Figure 5:**
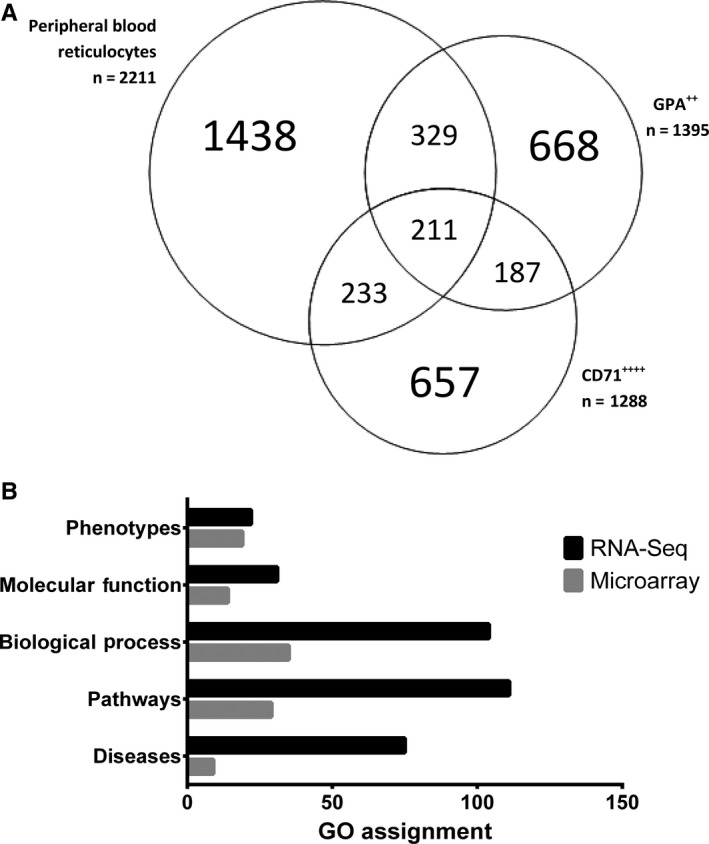
RNA‐Seq analysis. A, Comparison of the number of gene transcripts identified by our RNA‐Seq approach with the ones deposited in libraries representing the early (CD71^++++^) and late (GPA ^++^) reticulocyte maturation stages. Almost 35% of all identified gene transcripts in reticulocytes are identical to these deposited in libraries and approximately 30% of gene transcripts in both libraries are the same. Two hundred and eleven gene transcripts are common to the three groups discussed. Only transcripts successfully identified in all four individual RNA‐Seq analyses were considered. A detailed gene transcript list is provided in Supporting Information Table S6. Compare to Supporting Information Table S5. B, GeneAnalytics results. Comparison of reticulocyte transcriptome profiles obtained from RNA microarrays results previously published by Goh et al^21^ and our RNA‐Seq results placed in a physiological context was generated using the GeneAnalytics web server

The transcriptome profiles of the reticulocytes isolated from peripheral blood of hemolytic anaemia patients and healthy individuals’ reticulocytes were obtained with the RNA‐Seq method. The number of identified gene transcripts and their expression levels differ from the previous data obtained by the microarray‐based method in reticulocytes isolated from donors (eg, Goh et al^21^), as well as from data from cultured erythroid cells (GPA^++^ and CD71^++++^) deposited in the UniGene NCBI database: GPA^++^ library (Lib.11923) and CD71^++++^ library (Lib.8975) (Figure 5A).

As shown in Supporting Information Table S5, some of the erythroid gene transcripts present in our selection appear in late stages of reticulocyte maturation (eg, *SPTB*,* EPB42*,* DMTN*,* GYPC*,* GYPE*,* ADD1*). These data suggest that the differences in identified gene transcripts require further in‐depth analysis considering many factors affecting the gene profiles. Furthermore, it must be noted that the level of expression is regulated by factors such as gender, age, and even individual variations.

The ability to efficiently isolate human reticulocytes from peripheral blood should enable development of clinical diagnostic applications and facilitate defining specific defects underlying various human hematopoietic diseases. It needs to be stressed that due to the difficulty of obtaining pure reticulocyte population RNA, the data we have obtained on the reticulocyte transcriptome are among the very few available. Classic investigations of erythroid gene expression were usually focused on predefined sets of genes. The total RNA expression patterns of the purified reticulocytes from healthy individuals have so far been determined by RNA microarrays^21^ that are based on and thus limited to predefined probes. Goh et al detected 698 transcripts (488 named genes) in each of the 28 samples containing the total reticulocyte RNA. On the other hand, cell culture techniques permitted the isolation and functional characterization of erythroblasts at all stages of differentiation.^3^ The functional importance of most red blood cell proteins is unknown. To further explore the regulatory mechanisms of the specific erythroid gene expression and the function of the gene products during normal and stressed erythropoiesis, improved in vitro experiments need to be developed.

To test whether our reticulocyte isolation procedure combined with RNA‐Seq will result in improved coverage of cellular processes and their physiological/functional context, we placed previously published RNA microarrays results 21 and our RNA‐Seq results into a physiological context using the GeneAnalytics web server (Supporting Information Tables S3 and S4). As expected, the enrichment in identified transcripts in our RNA‐Seq analysis, when compared to microarrays results, was reflected in their much developed coverage of biological and functional categories. Briefly, transcripts identified in our approach allow convincing (high score) assignment of 111 metabolic pathways, 104 biological processes, and 31 molecular function pathways, whereas the microarray data were related to only 35 biological processes, 29 metabolic pathways, and only 14 molecular function pathways (Figure 5B). The changes in transcript levels can often serve as biomarkers for human pathologies, and in this context, data obtained by our procedure resulted in assignment of 75 diseases, while microarray data analysis allowed the transcript assignment to nine disease categories only.

Importantly, the extended list of identified transcripts obtained in our study provided much better coverage of gene ontology terms, as reflected by higher scores. Furthermore, using the GeneAnalytics web server we identified in our transcript set of genes associated with 22 phenotypes, whereas microarray data analysis resulted in identification of transcripts associated with 19 phenotypes (Table 2). Importantly, the genes identified by RNA‐Seq were associated specifically with erythroid phenotypes.

**Table 2:** Gene variants identified by RNA‐Seq analysis according to their expected associations with specific erythroid phenotypes. The set of genes identified in our human reticulocyte transcripts (RNA‐Seq analysis) using the GeneAnalytics web server was associated with 22 phenotypes compared with microarray data analysis^21^ resulting in identification of transcripts associated with 19 phenotypes (numbers in bold indicate high score, non‐bold indicates medium score)

Phenotype name	Genes related to phenotype	RNA‐Seq		Microarray	
		Score	Matched genes	Score	Matched genes
Complete embryonic lethality between implantation and somite formation	248	**30.09**	64	‐	‐
Abnormal erythrocyte morphology	81	**23.96**	29	**25.71**	14
Abnormal erythropoiesis	105	**21.08**	32	**16.28**	12
Spherocytosis	12	**19.61**	10	**20.66**	6
Abnormal erythrocyte physiology	22	**19.31**	13	‐	‐
Hemolytic anaemia	26	**19.08**	14	**17.89**	7
Increased mean corpuscular volume	76	**18.84**	25	‐	‐
Reticulocytosis	65	**17.49**	22	**20.36**	11
Anisocytosis	42	**17.21**	17	**23.09**	10
Abnormal respiratory electron transport chain	30	**16.77**	14	‐	‐
Anaemia	214	**16.59**	47	**21.18**	20
Complete embryonic lethality	297	**15.96**	59	‐	‐
Decreased embryo size	450	**15.93**	81	‐	‐
Microcytosis	20	**15.73**	11	‐	‐
Pallor	163	**15.63**	38	**22.67**	18
Decreased haemoglobin content	128	**15.36**	32	**17.94**	14
Decreased erythrocyte cell number	141	**15.15**	34	‐	‐
Complete embryonic lethality during organogenesis	540	**14.87**	92	11.46	27
Abnormal erythrocyte osmotic lysis	26	**14.53**	12	**14.37**	6
Decreased haematocrit	153	**14.04**	35	**17.20**	15
Abnormal cell physiology	261	**13.53**	51	‐	‐
Decreased mean corpuscular haemoglobin	57	**13.47**	18	**16.23**	9
Decreased mean corpuscular volume	59	12.86	18	**24.83**	12
Abnormal mitochondrion morphology	54	12.79	17	‐	‐
Poikilocytosis	30	12.62	12	**16.56**	7
Embryonic growth retardation	404	12.49	70	**16.91**	26
Abnormal haemoglobin	13	‐	‐	**29.21**	8
Abnormal mean corpuscular haemoglobin	5	‐	‐	**16.97**	4
Abnormal mean corpuscular volume	4	‐	‐	12.84	3
Hyperchromasia	5	‐	‐	11.90	3
Increased number of Heinz Bodies	6	‐	‐	**15.95**	4
Polychromatophilia	32	‐	‐	**22.97**	9

Taken together, gene ontology analysis supports the conclusion that the experimental approach proposed in this study provides an improved insight into biological processes that govern reticulocyte maturation, function and pathology. Undeniably, obtaining depth and reliable insight into related transcriptome profiles should significantly help to understand these mechanisms.

In conclusion, we have presented an efficient procedure of reticulocyte isolation from peripheral blood. This procedure should be useful in particular for normal, healthy individuals, whose blood is poor in reticulocytes, and for hemolytic anaemia patients including HS patients with increased hemolysis. This will increase the statistical power to detect disease‐relevant signatures in patient/healthy individual studies. Furthermore, obtaining reliable normal and pathological reticulocyte transcriptome profiles should contribute greatly to our understanding of the molecular bases of terminal erythroid differentiation, haemoglobin switching and iron metabolism, as well as the mechanism of hematological diseases, prognosis and responses to therapeutics.

## CONFLICT OF INTEREST

The authors declare that they have no conflict of interests concerning the contents of this article.
