# Supplementary material for: Efficient method for isolation of reticulocyte RNA from healthy individuals and hemolytic anaemia patients
Source: J Cell Mol Med. 2018 Nov 18;23(1):487–96. doi: 10.1111/jcmm.13951 (PMC6307756; doi:10.1111/jcmm.13951)
Supplement: Supplementary file 1 [file JCMM-23-487-s001.doc]

**Supporting Information**


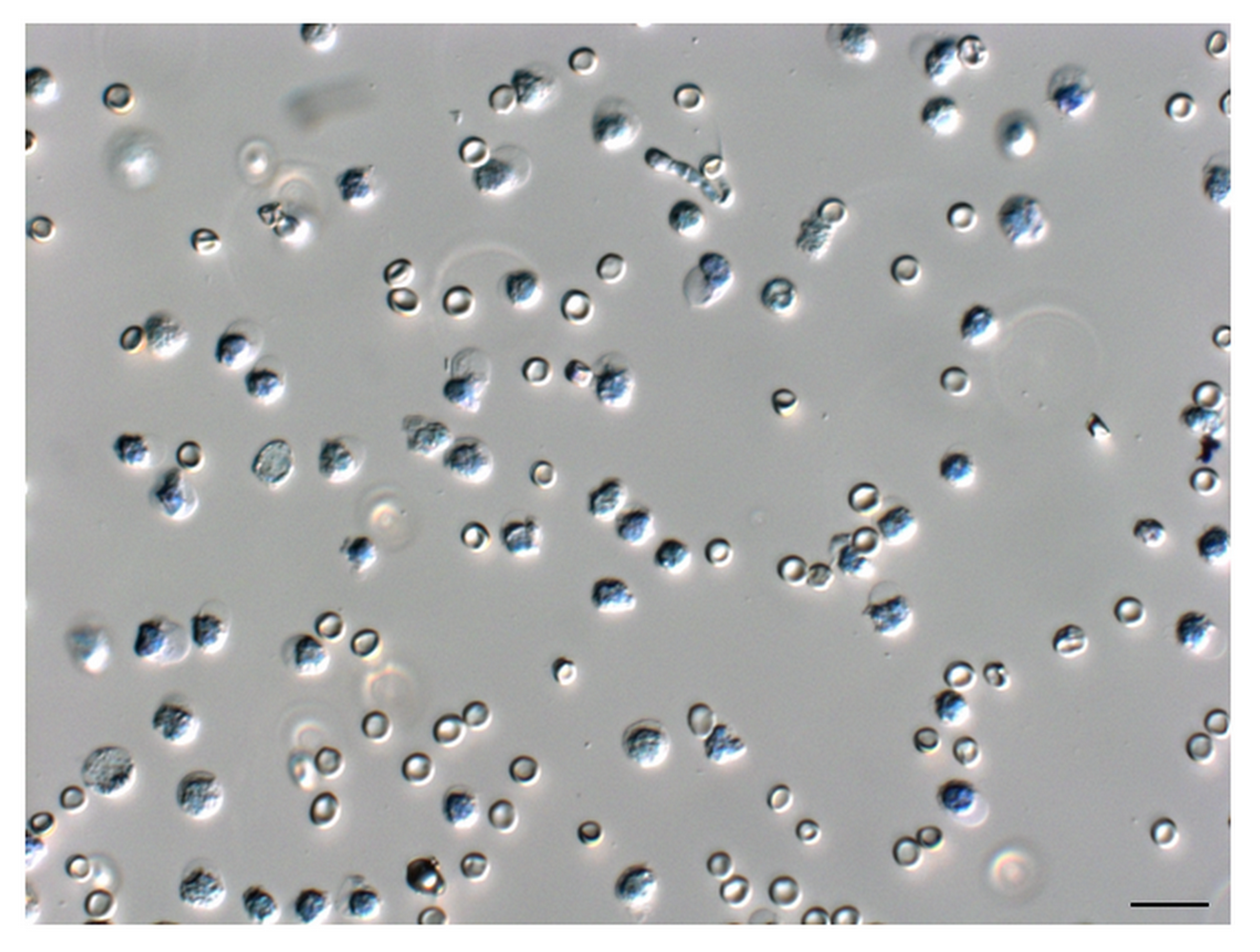
 Figure S1 Final reticulocyte-rich preparation under Nomarski phase optics (DIC). Reticulocyte were stained using brilliant cresyl blue. Red blood cells are unstained. Scale bar – 20 μm.

***Assessment of purity of isolated reticulocyte population by RT-PCR***

In addition, the presence of an additional transcript identified only in the leukocyte transcriptome, the transcript of the *ITGAL* gene (integrin subunit alpha L), which also gave a negative result (example data for patient C14, Supporting Information Figure S2) was tested. *SPTB* (spectrin-β) primers were used for determining the genomic DNA contamination (example data for patient C14, Supporting Information Figure S2). A sensitivity test of primers used for negative reticulocyte RNA verification was also performed on cDNA obtained from whole-blood-RNA as a template (data not shown). Primer sequences are reported in Supporting Information Table S2.


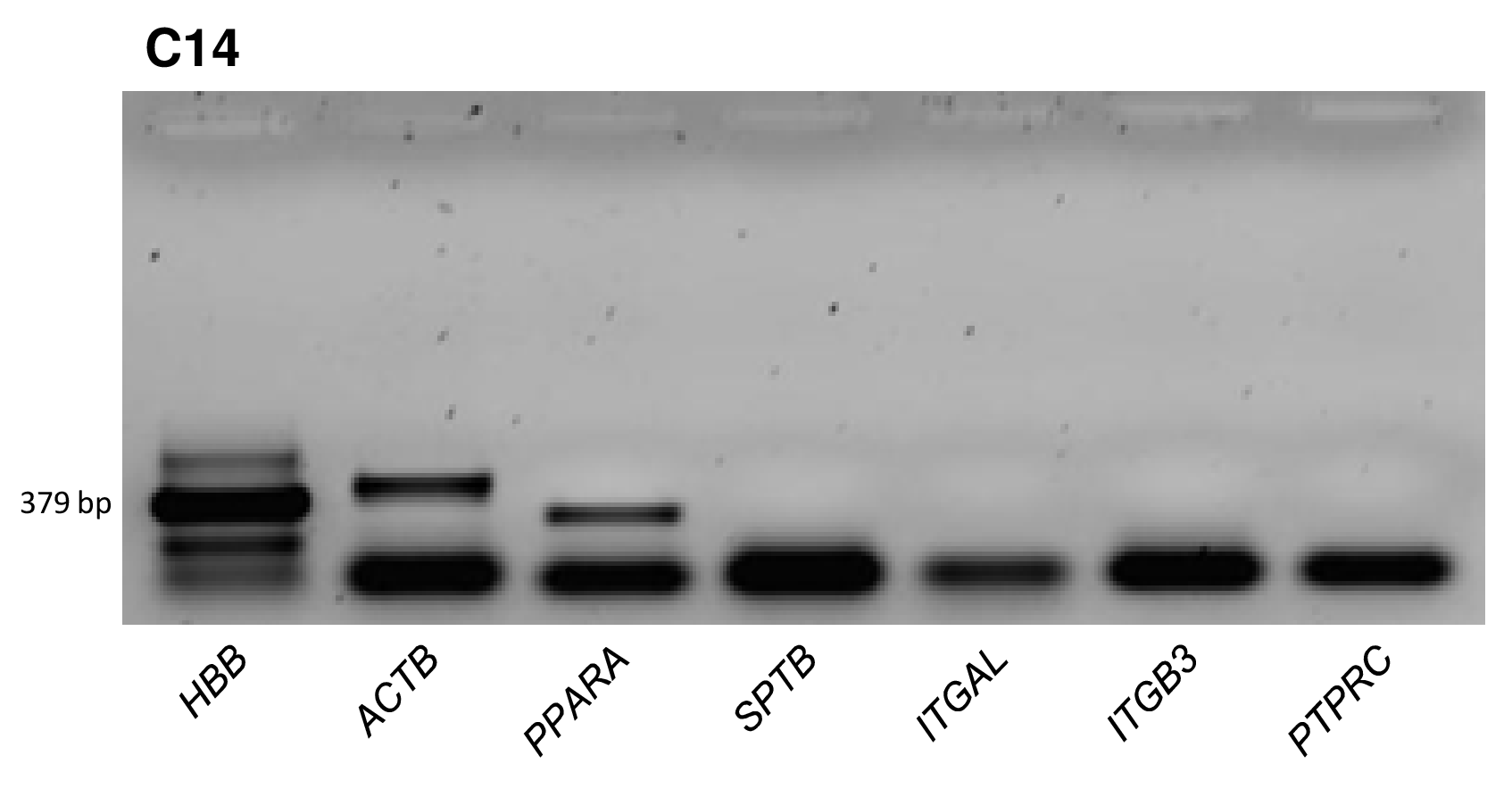


Figure S2 Reticulocyte cDNA quality control for sample C14, HS patient. Agarose gel electrophoresis of RT-PCR products (image inverted, black/white) obtained using, as with the other samples, primers encoding sequences of the following genes: CD45 (*PTPRC* gene) – leukocyte marker; β-globin (*HBB* gene) – erythroid marker; integrin-β3 (*ITGB3* gene) – platelet marker. As the “low abundance” gene transcript *PPARA* (peroxisome proliferator activated receptor alpha) was chosen. For the loading control β-actin primers (*ACTB* gene) were used. Additionally, *SPTB* (spectrin-β) and *ITGAL* (integrin subunit alpha L) primers were used to determine the genomic DNA and leukocyte contamination, respectively. Results show no genomic DNA, leukocyte or thrombocyte RNA contamination for all analyzed cases. As a standard "Perfect 100 bp DNA ladder" (EURx, Gdańsk, Poland) was used. The lowest band corresponds to the primer.

**Supporting Tables**

Table S1 Hematological characteristics of the studied healthy individuals (controls), HA patients and HS patient (all are male).

| **Healthy individuals/patients** | | **Age (years)** | **RBC  [*M/µL*]** | **HCT**  **[*%*]** | **Hb [*g/L*] *(remark)*** | **Total bilirubin**  **[*mg/dL*]** | **Ret**  **[*%*]** |
| --- | --- | --- | --- | --- | --- | --- | --- |
| **References for male** | | Adults | 4.2-6 | 40-54 | 140-180 | 0.3-1.2 | 0.5-1.5 |
| **Controls** | **C1** | 25 | 5.2 | 47 | 170 | 0.9 | 0.6 |
|  | **C2** | 30 | 4.9 | 43.2 | 144 | nd | 0.9 |
|  | **C3** | 37 | 5.4 | 47.2 | 157 | 0.8 | 1.0 |
| **HS patient** | **C14** | 39 | 3.95 | 36 | 122 | 3.08 | 7.8-14.5 |
| **HA patients** | **N61** | 40 | 3.3 | 34.9 | 110 | 2.0 | 3.6-7.9 |
|  | **N62** | 33 | 3.3 | 28.9 | 91 | 5.25 | 5.4-8.7 |

Table S2 List of primer sequences used for assessment of the purity of isolated reticulocyte cDNAs by RT-PCR (for *SPTB* gene).

| Primer name | Primer sequence | Target length |
| --- | --- | --- |
| HBB-F | 5’- GAGGAGAAGTCTGCCGTTAC -3’ | 397 bp |
| HBB-R | 5’- CCACACCAGCCACCACTTTC -3’ |
| ACTB-F | 5’- TACAATGAGCTGCGTGTGGCTCCCG -3’ | 479 bp |
| ACTB-R | 5’- AATGGTGATGACCTGGCCGTCAGGC -3’ |
| PPARA-F | 5’- CGATTCGACTCAAGCTGGTGTATG -3’ | 324 bp |
| PPARA-R | 5’- GATTGTTACTGGCCTTTCCTGAGAG -3’ |
| PTPRC-F | 5’- GACATCATCACCTAGCAGTTC -3’ | 217 bp |
| PTPRC-R | 5’- GCCCTGTCACAAATACTTCTG -3’ |
| ITGB3-F | 5’- CTGAAGGAGAATCTGCTGAAGG -3’ | 198 bp |
| ITGB3-R | 5’- CCGCACTTGGATGGAGAAATTC -3’ |
| ITGAL-F | 5’- GTTACACCGTGACCTGGCTG -3’ | 275 bp |
| ITGAL-R | 5’- GATAAACACCCGGCCTCCTC -3’ |
| SPTB-F | 5’- CTCCTTGCCAAGCACCATCCAGG -3’ | 398 bp |
| SPTB-R | 5’- AGTGAGGACGTAGCCTGCTCTGC -3’ |

Table S3 Gene set ontology results. Analysis of our RNA-Seq reticulocyte gene expression profile was carried out using the GeneAnalytics web server. The gene nomenclature was adjusted to the webserver requirements (GeneCards) updated to the current nomenclature.

Link: <https://1drv.ms/x/s!Ar7xJ2srxC-Pga1N9NSNbvhI0TTZXw>

<[https://1drv.ms/x/s%21Ar7xJ2srxC-Pga1N9NSNbvhI0TTZXw](https://1drv.ms/x/s!Ar7xJ2srxC-Pga1N9NSNbvhI0TTZXw)>

Table S4 Gene ontology analysis results. Analysis of microarray reticulocyte transcriptome profile (Goh et al. [[21]](#_ENREF_19)) was carried out using the GeneAnalytics web server. The gene nomenclature was adjusted to the webserver requirements (GeneCards) updated to the current nomenclature.

Link: <https://1drv.ms/x/s!Ar7xJ2srxC-Pga1Le99yxKFb6fZwVQ>

<[https://1drv.ms/x/s%21Ar7xJ2srxC-Pga1Le99yxKFb6fZwVQ](https://1drv.ms/x/s!Ar7xJ2srxC-Pga1Le99yxKFb6fZwVQ)>

Table S5 Erythroid gene transcripts with **successful and negative** expression status obtained from RNA-Seq data compared to gene transcripts’ presence in the data deposited in the UniGene NCBI database: CD71^++++^ library (Lib.8975) and GPA^++^ library (Lib.11923), respectively.

| Erythroid Gene Name | CD71^++++^ library | GPA^++^ library | Reticulocyte transcriptome (RNA-Seq) |
| --- | --- | --- | --- |
| ***ACTB*** | **+** | **+** | **+** |
| ***ADD1*** | **-** | **-** | **+** |
| ***ANK1*** | **+** | **+** | **+** |
| ***DMTN*** | **-** | **-** | **+** |
| ***EBP41*** | **+** | **+** | **+** |
| ***EPB42*** | **-** | **-** | **+** |
| ***GYPA*** | **+** | **+** | **+** |
| ***GYPB*** | **+** | **+** | **+** |
| ***GYPC*** | **-** | **-** | **+** |
| ***GYPE*** | **-** | **-** | **+** |
| ***MPP1*** | **-** | **+** | **+** |
| ***SLC4A1*** | **+** | **+** | **+** |
| ***SPTA1*** | **-** | **+** | **+** |
| ***SPTB*** | **-** | **-** | **+** |
| ***STOM*** | **+** | **+** | **+** |

Table S6 Detailed gene transcript list. Data from this study, cultured erythroid cells (GPA^++^ and CD71^++++^) deposited in the UniGene NCBI database: GPA^++^ library (Lib.11923) and CD71^++++^ library (Lib.8975). The gene nomenclature was adjusted to the webserver requirements (GeneCards) updated to the current nomenclature.

| **Name of analyses** | **Number of matched genes** | **Names of matched genes** |
| --- | --- | --- |
| List of gene names common for:  CD71^++++^ (1288), GPA^++^ (1395) and peripheral blood reticulocytes (2211) | 211 | *CNBP SLC4A1 COX7A2L ATP5F1 C9orf78 SUMO1 DDX5 UBB SOD2 RPL7A ARL6IP1 KPNA2 SUPT4H1 GNAS MYL12A NPC2 BUD31 TPI1 TPM3 HSPD1 NPM1 CCT8 EIF3M LDHB BTF3 SARS H3F3B RPL32 UBE2L6 PCCB HBG2 RPL4 OPA1 RPL10 YIPF6 EIF4E2 TPT1 MOSPD1 PARK7 MPC2 NOP10 TAF9 RHCE NHP2L1 UBE2D3 SLMO2 PSMD2 GYPA NACA CALCOCO2 SNCA BSDC1 RPS4Y1 RPLP2 CALM2 STMN1 YBX1 VPS29 NUTF2 H3F3A RPLP0 SRP14 USP15 SRSF7 RPS6 GHITM HNRNPA2B1 PSMB7 UBE3B EEF1D SOD1 MCM7 RPS3A RPL34 SSB DNAJC8 CPEB4 RPL3 TAL1 TMBIM6 PSMB4 PPP1CB HSP90AB1 PSMD10 SOX6 TMEM14B LUC7L3 EIF1AY RPS27L RNF10 BNIP3L XRCC5 NUDT4 CD164 RPL23A MMADHC RPS25 ZNF207 EIF3I MORF4L1 ZC3H15 PSME2 BMP2K RPL14 HNRNPA1 SAT1 BRWD1 HMGB1 OAT NDUFA6 NOSIP RPL36A PRDX6 PSMA3 PSMA1 SCOC RSL24D1 CHMP5 SKP1 SRI SLC25A5 CD36 ARF4 CSDE1 RPS4X RPS3 ARF1 RHOA C11orf58 RPSA TUBA1B FTL MRPS7 VBP1 STOM HIGD1A NAA50 RHAG HBD CDC26 SNX2 CINP EEF1B2 TFRC RPL6 NSUN3 H2AFZ SPCS1 UROS SRP9 PDCD10 RAN UFC1 PSME1 EEF1A1 HNRNPC HSP90AA1 PARP1 PSMA2 MRPL18 EPB41 SELK UROD SLU7 CD59 POMP ATP5O SUMO2 RPL21 ATRAID RPL5 GLUL GYPB GNB2L1 PCM1 PSMA7 FGFR1OP2 CA2 ANK1 PSMB5 COMMD3 SHFM1 ATP6V1G1 EIF3L PRDX2 ACTB EIF2AK1 B4GALT3 RPL13A RPL41 RPS7 MAD2L1BP HEMGN SERINC1 CCT5 PSMD7 EIF3K COX4I1 PSMB1 ATP6V1D CLIC2 SRSF5 SLC25A38 CA1 PDHB ATP6V0E1 AHSP TTC1 EEF1G DPCD RPS27* |
| List of gene names common for:  GPA^++^ (1395) and peripheral blood reticulocytes (2211) | 329 | *CD44 TKT DDB1 DCAF5 XK RNF14 MED13L ECH1 TANGO2 ARL4A ACTR10 DGUOK WDR45 EIF1 CETN3 USP7 YME1L1 RSRC1 LUC7L2 BRK1 CRIPT EZR ARIH1 POLDIP3 ASNSD1 NBR1 HSPA8 MARK3 ARPC3 RSU1 UBE2N PSMF1 CYSTM1 UIMC1 GPBP1L1 ETFA FTH1 CTSB TCP1 YPEL5 PSMC4 EIF3D RNF7 TANK MAP1LC3B DCAF11 ISCU THOC7 FDFT1 TALDO1 B2M AP2B1 CCNDBP1 KIAA0232 NUB1 UBE2K ERH FAM96A ATP6V1E1 BIRC2 ISCA1 SRSF3 BROX EIF3H ATG3 FAM211A FBXO7 GCLC ERGIC3 BLVRB GALNT10 BANF1 HMBS BSG GPBP1 CCT7 INPP5K PDZK1IP1 PSMD9 BPGM SIAH2 ABHD5 ATP5A1 IGF2BP2 MYL12B IFIT1B CHMP2A CR1L RAD23A CTNNA1 NARF APOPT1 DCAF6 SPOP RAB2B DAZAP2 SDHAF2 PIP4K2A PCNP RPL15 TRAK2 LGALS3 LPIN2 TIMM23 RPL28 IARS TSPO2 UBE2H USP33 SPTA1 PCMTD1 HIST1H2AC SSR2 COPS3 SYF2 HIST1H2BK CLCN3 MTPN MBNL3 MSMO1 SEC62 ZNF143 ENO1 FECH ARG2 SEC11A ZFAND5 C14orf119 ALKBH5 GDE1 TAB3 CAT CPOX NONO CDKN2C USP12 TMEM50A ZMAT2 RABGAP1L HADHA RPL8 CFDP1 SF3B14 PCBP2 FAM46C PIP5K1B TSTD2 ALAS2 ORC4 PSMA6 CYB5A MKRN1 ANXA7 NRD1 TMEM56 NARS YPEL3 KAT2B LTA4H PNP PGK1 MYL4 RAP1A SLC25A37 GABARAP TRIM23 SDHB EIF3B C20orf111 LAPTM4A PAIP2 SQSTM1 ARHGEF12 MAN2A2 NAPG RHD UBE2F BTG1 IKZF1 UBE2I DNAJA4 PCMT1 RNASEK R3HDM4 FOPNL KIF5B CCT2 DCAF12 HSD17B11 IDH2 SH3GLB1 COPZ1 GPCPD1 BCAP31 PTGES3 PRDX1 TBC1D22B NECAP2 ACTR1A ARMC8 NEAT1 EMC3 DCTN3 C12orf29 HECTD4 PIM1 TARS SH3BGRL PITPNA RPS2 FBXW4 ILK MTHFD2 AP2M1 NPRL3 MORF4L2 ALG13 YY1AP1 RTFDC1 LARP1 TMEM57 BZW1 CLDND1 RPL22 SLC38A2 MPP1 TMUB2 DNAJA1 ARG1 CDC42 NINJ2 MTIF3 RBX1 TXNL1 PPIB CCDC127 C20orf196 PNRC1 TMEM183A MAT2B VDAC3 SGMS1 FKBP1B DNAJB6 RPL23AP7 JAZF1 ATF4 TSPAN5 TXNIP TRIM58 KCTD20 TAX1BP1 KLF1 NDUFV3 ST3GAL2 TUBA4A CHCHD3 HIST1H3H GLRX3 EIF1B COPS5 SH3BP5L METTL9 SERPINE2 SP100 SDCBP ICAM4 CTSL1 HCCS EIF4A1 MXI1 GSTK1 DCUN1D1 CYB5R1 TBPL1 PSAT1 EMC4 CCNG1 ABCB10 ANAPC16 MICAL2 HECTD3 SNRPN C1D KIAA1191 FAH SNX3 RAB13 APOBEC3C RRP12 RAD17 SRSF9 RSAD2 LOC100127983 CTNNAL1 HTRA2 RAB11A GSTO1 ACSL6 HNRNPA3 CETN2 LSM1 DAP EIF4A2 PABPC1 TCP11L2 EIF2S2 HEPACAM2 RNF19A VTI1B LOC100506233 FOXO3 WNK1 KPNA6 SLC14A1 NPL GABARAPL2 DDX3X CRKL ANP32B DLEU1 NCOA4 POC1B SELENBP1 TMED4 AMD1 ATG14 GARS UBC* |
| List of gene names common for:  CD71^++++^ (1288) and peripheral blood reticulocytes (2211) | 233 | *RPS11 BRIX1 GAS5 RPL19 NDUFAB1 COX6C ANP32E DAD1 RPL35 NUDT1 MFF TIAL1 CCAR1 YWHAQ PPM1A RPLP1 PTP4A2 TRAM1 TMEM59 COX7A2 TMEM14C HP1BP3 RPS21 MRPS24 SNHG9 C6orf62 LSM3 CCT4 BRD4 NDUFB8 ITGB1 SERP1 HYPK RPS29 COX7B HNRNPK IK RPL39 SNRPG RPL17 HBQ1 UQCRQ MDH2 ABRACL USMG5 SF3B5 WDR26 CCDC23 RPS24 RPL27 RPL24 ZFAND6 PANK3 ARGLU1 PTTG1 MRPL22 NAP1L1 NDUFB3 CCDC167 COPS6 HNRNPH1 KPNA4 NUCKS1 ATP5J2 EIF5B HMGN2 CORT UQCR10 GADD45A RPS10 ATP5G3 STX18 PFDN1 RPL18A UBL5 NUDC SYNCRIP ATP5J TUBA1C TXNRD1 CYBB CCT3 NDUFA1 HSPE1 NCL RPL9 IRF2 PPP2CA NOL7 TM9SF3 DCAF10 TOMM5 ARL6IP4 RPL35A CBX3 ANKRD13A ACP1 SRA1 KIAA1586 MRPS21 RPL37A SERF2 RPL11 RBM39 FAM104B OIP5-AS1 NDUFB2 WDR83OS UBE2V1 EIF3E RPL31 PSMB3 RPN2 OST4 COX7C C14orf2 HINT1 HNRNPU RPL38 RPS16 CSNK2A1 CALCOCO1 TMEM256 PPP1R7 UQCRH ACTR2 CDYL RPL10A C19orf53 STRADB ILF2 AIDA RPS23 ATP5I DAP3 TMEM66 MGST3 RPL12 JTB PSMB6 SLC40A1 POLR2I RPS27A UFD1L SLC1A5 RPS13 MYL6 RPS8 ARIH2 PPP1CC ZC3HAV1 YY1 CKS1B STUB1 RPL7 COPS2 MRPL20 NDUFA4 SLIRP HK1 TPM1 STRAP RFESD PPIA SEC22B OCIAD1 PFDN5 RPL30 ADRM1 DYNLRB1 ALDOA NUDT3 RPL36AL EIF4B RPS15 PTMA RPL26 OSTC RPL36 ANAPC11 ATP5L OPTN TOMM6 AHSA1 NME2 S100A4 RPS20 RAP1B EIF5 SAP18 HSPA5 RPS12 TMEM245 TSG101 HSP90B1 SNHG8 TIMM8B RPL27A FBL PET100 COX5B NEDD8 INPP5D CDC16 RPL23 PSMD1 LINC00152 LRRFIP2 DLD PAICS PHF20L1 TCEB2 TMEM258 NDUFS5 PFN1 MICALL2 KIAA0430 UBR2 HDGF TXN MAP4K4 TMSB4X SSBP1 ATP5E REXO2 SERBP1 TOMM7 RPL37 TMA7 HBG1 NDUFB9 TOP1 SNRPD2* |
| List of gene names common for: CD71^++++^ (1288) and GPA^++^ (1395) | 187 | *PDHA1 ABCB7 CYCS THRAP3 ING3 TTC37 RCL1 ANLN TUBB MAP3K13 SRSF11 SCP2 XRN2 PSMD11 HPS4 QRICH1 FUBP1 DDX24 BCAP29 MTHFD1 TIMM17A LXN TLK1 RBM26 LOC100508408 EIF2S1 SDHD PLRG1 FKBP5 GIGYF2 DNA2 MRPS9 TMEM106C SCFD1 SAR1A MRPS18C SSBP2 UBE2T BUB3 UBR1 COX16 EIF4E TFAM SLC31A1 HAUS1 SP110 WDR33 TACC1 NUPL2 EIF3A FRG1 MRPL13 CMC2 CCNB2 PRC1 NAA38 UFSP2 MPHOSPH6 ARL6IP5 ANKRD36 SNAPC5 MRPS31 DAXX ZCCHC17 GTF2B CDK7 PMPCB MED21 DCAF13 HBB RAD51AP1 MKI67 AGPAT5 HILPDA AZI2 SARNP ATG12 HAX1 SNRPB2 KIF22 ANKRD32 BBIP1 SBNO1 TBC1D23 SUMO1P3 MRPS18B UBR4 USP8 ZNF17 ZNF22 IFIT3 EIF6 CHMP4A RC3H1 FARSB ASCC3 MDH1 CNOT4 THYN1 PSMC2 ANAPC13 USP16 NPAT ZMYND8 TSC22D3 PRKAA1 EXOSC9 PSMC6 MAGT1 FAM188A AASDHPPT PPHLN1 CNTF SMARCA5 PCNA EPRS DDX52 THOC1 UBA3 ATXN3 MRPS22 CENPN NXF1 ARHGAP19 DDX46 MLLT3 GOSR1 EXOSC8 PSMC1 CEP95 CLTC HMGB2 ECI2 SUCLG1 PRDX3 NIPSNAP3A LRIF1 SMC2 EXOSC1 ARHGAP18 PRIM1 EAPP POLR2K ORMDL2 CDH1 NEMF ACAT2 RPAIN COMMD10 CAPRIN2 CRBN TTF1 TIMMDC1 PAFAH1B2 MRPS27 MRPL40 KIF18A SELT RRM1 TMX1 RBMX MDN1 BCCIP HBS1L CACYBP TRAPPC4 RBM8A NDUFA9 YBX3 MALAT1 RAD21 SEP15 CEP152 ATP5C1 HIATL1 AKR1C3 UBE2V2 YTHDC1 CAST VTA1 SCAF11 CDKN3 CUTA FDPS CENPF NUSAP1 HBA2* |
| Unique gene names for peripheral blood reticulocytes (2211) | 1438 | *DPM2 FSTL1 ACTN4 KRTCAP2 PTPLA PINK1 IFT52 NDUFS6 ERAL1 VPS4A HSPB1 DDT GFOD2 ARRB2 DECR1 SLC25A3 RMRP HERPUD2 TMSB10 HSD17B10 G3BP1 RAB1B UBE2G1 AMZ2 UQCR11 UBAC1 GDF15 DAZAP1 CCNI TPRG1L GZMM PAIP1 ATP5G2 SLC38A5 RPPH1 ARRDC2 AES ARL2 SYCP3 PAPOLA STK16 MAX ATP5D RPS5 PI4KB ZFYVE1 OAZ2 HMGN1 TMEM184A POLR3K C20orf27 MAPKAPK2 ATXN10 LHFPL2 FAHD1 DIP2A SHC1 BLOC1S2 CMPK2 FAM96B ITM2B ACSM3 KRT1 MT-ATP6 VKORC1 SLC25A46 TERF2IP CLTA COL1A1 PDCD5 GTPBP1 PDIA3 NT5M GDPD5 CIDECP CCDC124 MT-ND4L GOLGA3 TRA2B EXOC3 RAD23B STK17A MGAT4B C19orf43 CTDSP2 WDR59 SSU72 LAMTOR2 WHSC1L1 USP14 DSTN GYPC GMPR CDK8 IP6K1 ZNF598 PIN1 EPB42 RPS28 C17orf99 ADH5 FABP5 DESI1 CDIPT GLTSCR2 RFX3 CD82 SPPL3 EIF1AX HBZ ADI1 MAFK DENND1A PLCL2 SEC14L1 CHFR PSMD3 RHEB TBC1D17 COX8A TSR3 C9orf16 BCAM PCK2 CHST11 ALAD POLR2J UQCRC1 TFR2 UPK3BL TSPAN7 MFN2 NUCB1 PTMS LRRC8A DDX49 RAB5B IRF3 TAF10 CAPN1 GBGT1 FAM204A APOBEC2 MAPRE1 TRIP12 NDUFA2 DOHH BACE2 METTL21A USP32 PRDX4 LSM12 KDM4B NTAN1 CST3 NUP88 CSDA MOB4 NDUFC1 ARL2BP TMEM11 NQO2 UQCRHL PICALM CNOT6 DRAM2 STK40 TSN GSTP1 SUV420H1 AGTPBP1 PRR5 MGST1 FKBP4 PNPLA6 C11orf68 FAM108A1 MT-ND2 ANXA2P2 YIPF2 MYEOV2 GATA1 PBX1 UBL7 VAMP4 RBM38 ACO2 LOC150381 C5orf4 LSMD1 HNRNPUL2 HEXIM1 YWHAZ IFI27 MT1G MOB1B IDS USP4 SFRP2 RNF182 PDAP1 NDUFA13 MRPL41 YWHAH FAU AKIRIN1 FGD5-AS1 JUND ITSN1 PHB2 EHBP1L1 CYHR1 SIRT2 ERMAP C1QBP GANAB ASAP1 TMEM248 HNRNPA0 PGAM1 IKBKG PCBP1 NGFRAP1 NEDD4L HAGH PLK1S1 MTRNR2L8 ERCC1 VWCE AGO2 ASTN2 WBP1 RER1 TUBA1A TAB2 DDI2 KIAA0930 HPS1 TFE3 ZNF511 LOC440288 PSMD8 UBALD1 SET DNAJB4 THY1 TMEM189 C17orf89 FAM120A KRT8 AURKAIP1 ATP5H SLC50A1 SVIP RFNG PPOX EPB49 RAB5A ACADVL MED25 ZBTB8OS SPP1 PHGDH GPS1 WIPI2 RANBP1 TREML3P LAMTOR5 HCFC1R1 SEPHS1 EIF5A RMDN3 CTNNB1 TRAPPC5 TPGS1 CLK3 FIS1 TGIF2 KDELR1 VPS16 CCND1 LOC100505817 DNAJC25 ADD1 FAM122A E2F4 POP7 C7orf41 SMS GPX4 SRGAP2C PRMT1 CHMP3 CCDC176 PEF1 PPDPF ZFAS1 PAGE2 IBA57 FAM122B RABAC1 SLC48A1 DNAJC4 CDK16 PAPD4 MEF2B PTPN4 STX5 RCN1 TXNL4A SRRD CRAT HN1 SDR39U1 PPP6R2 MT-TC RNF175 UXT AP2S1 SENP3 SLC5A3 CHST2 KCTD2 FRMD4A TOMM22 TMEM259 S100A16 TIMP1 TPST2 DPY19L2P2 MIA MT-ND6 EPN1 VCP FAM120AOS RCC1 SH3GL1 AUH SLC25A39 RAB4B ABHD14B MT-TS1 MRPL37 NDUFV2 KLHDC2 SMIM1 ALKBH7 AMPD3 FAM104A SNX29 SNURF PYURF TMEM134 MMP1 LOC91948 ZNF3 ALDH5A1 SEC61A1 POLD4 MRFAP1 NME4 FBXO30 SLC2A1 CREM EPCAM MMP24 SSR4 CKS2 TOR1A UCP2 FN3K ARHGAP8 PPP1CA PCSK1N PABPN1 GBA2 FBRS SLC25A42 FKBP8 COX6A1 FAM83A NDE1 ANXA2 KLHDC8A AK1 STK24 EI24 CARHSP1 AAAS VPS26B DGCR8 GSR FAM20B ATP6V1F CCDC94 PTTG1IP GGH CDC27 DCTN4 PLBD2 ASNA1 WDTC1 HEBP1 PPP1R15A CITED2 MAN1A1 INS DYRK3 PPP2R4 PKIG INO80B HNRNPD NEIL2 MKNK2 TMBIM1 YWHAG CCS RMND5A URGCP TSSC4 MAF1 CCDC117 PPP3CB FLCN SSBP3 TBCEL MZT2B NDUFB7 ROMO1 MFGE8 C17orf49 PNMA2 RNF167 ACP5 AMFR THEM5 MAPK1 ATPIF1 RNF126 CLIC4 UTP18 PPP1R14B ANKH PPP3R1 PDCL FAM117A EID1 RBM14 SF3A1 CAPNS1 COPE SDF4 PPP2R5B KLHDC3 AKT2 ZDHHC3 ST13P4 GALNT6 ZNF271 POLL FXR2 FBXO9 SNX5 RAB6A PTOV1-AS1 FSCN1 CHD2 UBAC2 FZD5 ST6GALNAC4 FAM134A FLJ13197 IGFBP6 CREB1 MRPL55 POLDIP2 MXRA7 SLC37A2 DBI CFL1 MRPL14 PIGQ TAX1BP3 MINPP1 CLPTM1L BLCAP TTL ATP6V0C SLAIN2 PSMD6 TMC5 CTSE ITFG3 FKBP3 PLEKHB2 SLC35A2 SHISA4 AP1B1 DYNLL1 TBL1XR1 UBE2O G6PD LOC729013 EIF3J TRA2A PIK3R2 CMAS MAP2K7 NPIPL3 SP2 SLC6A8 CCDC91 MT-TY ARF5 SLC3A2 PPP1R9B GSPT1 LOC100132247 DNM2 RNPEP MRPS16 KRT18 KIAA0922 KCMF1 WWC3 TCEA1 ARPC4 EGLN2 C7orf55 MRPS36 CLPX NPLOC4 STAU1 CHMP1A C22orf32 MT-ND5 HADHB PSMD4 NDUFA7 HBM TNIP1 SHOC2 PSKH1 TMEM200B MED15 CYB5R3 FTH1P3 C1orf105 C21orf33 SNRNP35 CDC37 ZER1 MT-CO1 IDH3G SNX22 ODC1 CROCCP2 YIF1A GRINA ADIPOR1 DNAJB2 JAZF1-AS1 UBE2MP1 SNF8 H2AFV E2F2 BBS5 DDX17 C5orf27 TMUB1 EMC6 PQLC1 NT5C3 SBDS AKAP8L RAP2A ATP6AP2 AZIN1 NDUFB11 OSBPL2 C16orf58 ASH2L KANK2 RNF139 C18orf32 SMG7 SMIM5 TMEM115 STK11 PRPSAP2 OSBP2 SH3BGRL3 CHP1 EXOSC4 C7orf73 ELOVL1 PDIA6 MGC2752 DCXR SUCO GUCD1 CXorf40B EHD1 MARCKSL1 C9orf69 CDKN1A LOC439994 SLC7A1 MRPS15 FAM214B JMJD8 TPM2 CPNE1 OLFML3 NREP MBP LOC285074 LZIC GUK1 FAM210B TRAK1 CSTB C22orf39 CTDNEP1 SNHG6 HSBP1L1 EIF4H MRPS6 C9orf40 ETFB ASXL1 TUBB4B SNHG1 MDP1 BMI1 PCTP VRK3 FASTK UBALD2 BOLA3 DUS1L UBFD1 GNB1 HIST1H2BD TIMM17B SNX15 RNF181 RFFL AFTPH APBA3 AGBL5 PFDN4 SLC25A51 DEDD2 LY9 KDELR2 RAB10 MRPL23 ABCC13 EXOC7 TXN2 KLF13 CSDAP1 ZFAND2A TOLLIP DEK TRAPPC3 UPF2 RNF11 PLVAP DEXI PHB MRPL36 CLTB C2orf88 WDR13 GAPDH IFRD2 SEC61B GPX1 PYCR2 LIN28A HIST1H1C GNA12 MAP1LC3B2 POLR2E HIST3H2BB TSPAN17 TRMT1L HIGD2A CYBRD1 MT2A MGRN1 FBXL4 LBH ZFAND2B MIEN1 ACTG1 NFU1 CALM1 RNF123 CHCHD2 MAP2K3 NME1 SMAP2 RPS9 BRAF APRT PEBP1 COL1A2 GPS2 RALBP1 HGS HRSP12 ABTB1 CCT6A SLC16A1 NDEL1 RANBP9 LAPTM4B TMEM147 YIPF3 XPO7 RRAGB CAPZB NKIRAS2 SOX2 NDUFA5 SDHA COMT HNRNPU-AS1 GNPTG C1orf43 RHBDD1 UQCRC2 RYBP SYAP1 RNPEPL1 SNORD49B HDLBP C15orf61 EIF2D FAM50A MLXIP KLF3 FZR1 PLOD1 KHNYN GPAA1 C17orf103 HYAL3 CHPT1 CLPB ACTR3 HECA EIF4EBP2 TBCB NDUFB1 CNPY2 DLGAP1-AS1 COX5A POLR2L TSTA3 RAC1 MOB2 NFE2 PCMTD2 TOMM20 ZDHHC2 PTEN ATP6V0D1 C17orf76-AS1 ROGDI DCK TFDP2 HMG20B PTRHD1 USF2 NDUFAF3 SLC22A4 C20orf24 ITLN1 SQRDL PTK2B KEL DNAJB1 CHID1 SERINC3 USP20 C18orf8 UBE2E3 GJA1 GNAI2 TAF7 SFPQ NGRN TGM2 MECP2 LAMP1 RXRA TMEM60 PDLIM7 GRK6 RPL29 VPS26A SLC44A2 STAG2 LOC730183 MT-TA CD55 NMRK1 SCYL1 PTOV1 YOD1 UCHL1 YIPF4 CDK12 NDUFA11 TAGLN2 ST3GAL1 THAP11 ATP6V0B PAFAH1B1 APEH MT-TN MAPKAP1 LGALS1 AKIRIN2 YWHAE WDR1 MRPL24 FAM89B LSM14A NDUFS8 RBM4 MXD4 PQBP1 CRYAB NABP2 COPA CKB RTN3 ULK1 TRMT112 GNG5 RPL13AP20 ESRG CLPTM1 PPP6R1 ATP5B SECISBP2 ZNHIT1 HMGN3 XRCC6 SUPT5H RAP1GAP GNG10 AGPAT3 CDK2AP2 KCNH2 HDDC3 SND1 C1orf123 LOC100129250 PI4K2A TEX261 ZFAND4 LOC541471 HIST1H2BC RNF145 GATAD2A ARHGEF2 PIH1D1 SWT1 RCOR3 TSC2 OR2W3 COX17 CASC3 GTF3A MRPL54 TMCC2 BCL2L2 LOC100129361 SNRPF SGIP1 MAL HIRA BAG1 GYPE CNN3 ORMDL3 PAN2 DHX40 NDUFB4 LSM10 GFI1B C12orf10 TUSC2 FADS2 TMEM230 MT-CYB POMZP3 RNF103 NRAS RDH5 ZNF410 TMEM165 FOXJ2 RPS19BP1 ABCC4 SCAND1 UHMK1 MPND TADA3 USP9X MEA1 PAGE2B EMP1 PFN2 MT-ATP8 TM7SF2 FLOT2 LEPR LTBP2 ARPC5 VPS51 CDKN2D NRBP1 CRYBA2 THTPA HIST3H2A LEPROT TXNRD2 DDX42 C11orf71 DYNLL2 PITHD1 TTC25 UGDH-AS1 MIIP EMID1 AP2A1 UBE2M FURIN UQCRFS1 GSN P2RX5 PLEKHA3 SNX4 NAT6 GPKOW SMOX SF3A2 MAP2K2 CGGBP1 FHL2 SUGT1 VIM CALR UQCRB SUMO3 LINC00853 RRAGA LMO2 ZRANB1 HLA-A JHDM1D CYC1 LDHA MGC72080 RWDD3 CMC1 FEM1A C1orf122 NR1H2 ZDHHC12 PDK2 SRGAP2 CD2AP GID4 RPS26 TMEM9B KXD1 TNK2 DENND4A MT-ND4 CAP1 TBC1D20 ASCC2 PSENEN BABAM1 STX7 PRPS1 MT-CO3 NAP1L4 CAMLG ATG4D RNF187 MRC2 RGCC MTCH1 SZRD1 BAD NAA10 LOC440434 SH3GLB2 ATF5 GRHPR CRTC2 MEGF9 LINC00570 GABARAPL1 EDF1 HNRNPUL1 ECSIT POLR3E C14orf166 RNF115 CISD2 SPECC1 GTDC2 TNS1 KLC3 CD9 PRDX5 EIF3F RALGDS ATG13 CTSD STAT6 RPSAP58 RIOK3 ZNF653 GID8 S100A6 ATG9A LHPP CRYGB ILF3 UBE2B MT1X BCL2L13 MED16 POLR3C GNB2 C12orf57 TMEM63B CSNK1A1 RNF5 MFSD1 RPS6KB2 LRP10 UBAP1 ANKRD9 MT-ND1 MAP4K5 ACHE HNRNPH3 KCTD9 FUZ SURF2 INO80C KPNA1 PRNP DARC RFXANK S100A11 MAZ CHMP4B GALNT4 SRSF8 PSME4 ANXA5 PTBP1 SSNA1 KARS H1F0 EPOR ETF1 EIF4G1 ING2 CCDC142 ISG15 CREBL2 RAB11B DYRK1B SCLY UBE2G2 CCRL2 YWHAB ENTPD5 MRPL43 GOLGA7 DNAJC6 TMEM86B ZNF213 BLOC1S1 WDR81 ARPC2 MFSD2B RTN4 GAPVD1 NAPA NDUFB10 IVNS1ABP PRKAR2B GLUD1 ABCG2 ZNF394 EEF2 RPIA RBM12 H2AFJ CRYBB2P1 NUP98 STXBP2 TMEM158 CARM1 COTL1 DEDD TMEM9 SCYL2 BRAP SESN3 ZNF580 TUBB2A YPEL4 UBE2S MOSPD3 PNRC2 CD24 ERP29 DNAJA2 PSMG3 PSAP HNRPDL CRYAA EPB41L4A-AS1 PLEK2 TPM4 MRPL3 ZNF428 IFI6 COX6B1 DNASE2 NOP56 SRXN1 UBE2R2 IGF2 NPEPPS DENR FOXO4 SNRPA1 PCGF5 MCOLN1 NFIX PPM1B SLC7A5 YBEY ZNF23 TPP1 HNRNPF TBCA SNHG5 CDK2AP1 DHPS TFDP1 MT1L TOX4 SURF1 TUBGCP2 RPS15A LOC100507217 FBXW5 METTL22 PPME1 RBM42 TRAPPC1 TSPO HES6 MBNL1 TPGS2 BRI3 HPCAL1 SPG21 MRPS5 CREG1 EMD WIPI1 CMPK1 RWDD1 NPEPL1 ZNF777 ENSA SAP30 PDZD8 SLC43A1 HNRNPM ENDOD1 SRSF1 IDI1 RPA1 VPS28 LAMTOR1 ZDHHC5 MDK MT-ND3 CD63 KLHL12 ZNF346 HNRNPAB SLC20A1 GADD45GIP1 UBA52 SPTB MICALCL CAV1 SGOL1-AS1 BCL2L1 C19orf77 RAB3IL1 LGALS9 USP34 APLP2 ELOF1 MSI2 GLYR1 WBP2 RPL18 ISG20 MLST8 POLR1D EPM2A GPR146 CD81 UBE4B CTDSP1 H1FX AP3D1 RPS19 OTUD5 LYL1 OR2T8 MED28 SNHG10 PTPRA STOML2 HSF1 DR1 BAP1 UBXN1 GLRX5 RAB5C TTLL3 GDI2 METTL7A WTAP MLF2 CRYGS NFKBIB AKT1S1 TSC22D4 CAPN5 MLLT10 NPIP PA2G4 S100A10 TDGF1 FAM3A MFSD12 RPL13 RILP ZFAND3 AQP1 SPSB3 KLHDC10 ST6GALNAC6 CALM3 SRSF2 B4GALT7 LOC100134229 BSCL2 TCF3 UBQLN1 RANBP10 NUP54 RNF40 TSPAN6 TMOD1 PRELID1 MRPL21 C19orf24 RGS6 UBXN6 CIZ1 GPR137 ZFYVE21 CIB1 MBD3 OXSR1 REEP4 CLEC16A RAD51D DYRK1A DRAP1 KIAA2013 POLR2J2 DNAJB12 PKM C19orf60 C6orf106 LOC100506963 TBC1D25 LY6E MT-CO2 SRGAP2D GCAT CDV3 AUP1 CORO7 NDUFA3 ZNF286B LYRM4 CANX RGS10 PPP2R3C CUL4A SHARPIN UGP2 CDC34 CIRBP SEC14L4 FOXP1 SLC6A9 MEF2BNB PTCD1 LOC728190 TMEM179B SUDS3 SIK3 SNRPB MIF ZBTB44 FAM65C ST13 CNPPD1 PSMC3 RARS LRRC28 P4HB RSC1A1 SAE1 CERS2 LOC256021 OAZ1 BLOC1S6 GSK3A ELL2 SPARC WWP2 ACTA2 DERL1 BECN1 ZNF592 FBXO18 EIF4G2 CTSZ STX16 HNRNPH2 KPNB1 ZNFX1 ARAF CRYBB2 DCTN2 CLNS1A IFT20 FUNDC2 MGEA5 ING1 PHOSPHO1 MFHAS1 TMED1 PPA1 ST7 HMGA1 TESC DCP1B APITD1 RPS14 C1orf116 MZT2A ESPN UBE2D2 MTMR3 PAM16 SDCBP2 HNRNPL UNC45A PGD RUNDC3A TADA2B NDFIP1 WAC ECD SFRP1 NANS FKBP1A EIF3G NDUFS7 GOLT1B SLC18B1 HIPK1 STK17B ADD2 MAT2A STX4 MBOAT2 CDKL1 MRPL53 SLC10A3 APP VDAC2 PHF21A WASF2 PNPLA2 UBE2J2 FAM21C HDAC6 SNX17 PFDN2 PLSCR3 RAB7A CBR1 DCAF8 SHISA5 CTSC MPST* |
| Unique gene names for GPA^++^ (1395) | 668 | *C5orf42 TRAF3IP2-AS1 SNORA42 DLG1 MT1B POLR2G GPR98 CLK4 HOMEZ STYK1 EP300 NOS2 BTN2A1 CCNB1 NDUFS1 TMX4 LOC100216546 MIER1 C1orf27 SEMA4D AP3S2 LOC100996307 HIST1H4E CASP6 PIR LRRN1 PIKFYVE ARF6 ATG2B FAM209A SAAL1 TAOK3 KIAA1033 PUM3 STX8 GINS1 BBS12 EED BIRC5 LOC100506098 WHSC1 RAPGEF6 PPIL1 BORCS7 KNTC1 GTF3C3 AP3M2 PSMG4 CDK1 N4BP2L2 PGA4 PLIN2 ZSCAN16 IFT140 C11orf73 MZT1 TAF12 PRPS2 FBXO24 DNAH11 GSTM4 DNAH12 CNOT1 PDCL2 SNX6 EXOC2 HSF2 SRP72 ZNF24 RANBP6 CDK17 INO80D ABI2 QKI OFD1 DYNC1H1 TMEM33 RGPD5 WDR20 WDR6 WDR62 ITFG2 GCA SLC22A16 CIITA ZNF79 GLA NGDN IMMT ZBTB20 BRD8 NR2C2 ASAH1 STMP1 HEATR5B FAM126B MAD2L1 ATP6V1C1 TTC21B SERTAD2 COQ10B ATP1A1 NRF1 SETD8 PGM2L1 DISC1 C20orf173 CCDC6 SEC63 MRPS28 PIEZO2 DTNB ADRB1 ALDH6A1 CHRNB1 UMPS LRRC2 PAQR3 PREX2 KLHL21 XRCC4 IL8 LOC153684 FBXL20 LMAN2L LAMTOR3 OSBPL3 METTL5 CORO1C HBP1 XIAP CSNK2B OLR1 MAP1A NELFCD TMEM69 ATP6V1A INPP5B GRPEL2 ORMDL1 RIC8A NOL11 GCNT2 TYMS KAT8 SIK2 DMXL1 CUL3 DLEU2 MSTO2P METTL16 ZNF677 LOC100506990 ZXDC ABHD18 ZNF530 C1orf131 NATD1 ART4 HIST1H2AG LOC729461 GTSE1 CGRRF1 PLOD2 NUF2 THEMIS2 FAM91A1 ZBTB4 MBD2 TSPAN31 ALAS1 PDE6G HMGXB4 GREB1L TRPV1 ATG4A ALS2 HSD17B13 ZDHHC17 HMGCR KDM4A CRADD COPB2 HMOX1 WDR61 MRPS10 RB1CC1 POLR3A LOC100507577 PCDP1 SLC35F5 YAE1D1 CNTLN IL17RD PCLAF TBCE HN1L TSC1 OARD1 ARL17B UGGT2 DHX8 IGKC SPTY2D1 PTPRF IKBKB SESN1 APPL2 DPY19L2P1 NECAP1 WDR74 USF3 TRAPPC11 APOH TSC22D2 SEPT2 METTL2A PRG2 ATF2 SP140 NOX5 TMEM167A ACVR1 GOLGA2 NIF3L1 HCFC2 PAAF1 PMAIP1 SLC7A11-AS1 GUSB ADCY10P1 MCMDC2 C10orf118 LRMP IFT88 IST1 GALNT14 FGF5 ANKLE1 GGCT NUMB PAN3 XRN1 SSR1 RHOJ NUP107 ZNF507 TMED10 DLGAP5 TFPI HAT1 MLL5 CA3 ADAMTS3 TGIF1 CLSPN PPP6R3 OTUB1 EMR3 ZNF438 TATDN3 CDC23 C16orf80 TBC1D15 ITCH FAM118B HIST1H4H PDGFRA CIAPIN1 HELZ LSM2 NFYB NFXL1 FNDC3B VMA21 FTSJD2 GFRA1 NEK9 USP50 C5orf22 RAMP2-AS1 TOP2B TLK2 C2orf47 MLH1 POU2F1 WDR83 ANKMY2 STX6 PLEKHA5 TSFM SGK3 GLB1 INO80 STAB2 MTCH2 USP39 DYNLT3 CWF19L2 RFC5 AKT3 IL1R1 SF3B3 C11orf70 ITGB5 NUDT21 LRRC75A-AS1 LOC100505876 TP53BP2 PDLIM5 BTAF1 NSUN4 RNASEH1 DNASE1 SLFN14 RAE1 TVP23C ELP4 NBN QARS MBNL2 GTF2A1 AGFG1 SETD2 LINC00308 UGGT1 ZNF473 SMARCC2 FAM234B SYNE2 CXorf24 CALB1 ZNF347 LYRM2 DDOST FBXO8 MGC15705 DDX19B RGS16 URB1 SLC25A26 CLN8 SMIM3 STAM FCGR2B LYRM1 RNF141 STX2 FUBP3 GABPB2 MPP7 FBN2 MLL DDX47 DPH3 RPL7L1 UBQLN4 CASP10 MUC16 RBM23 FAS SLC2A1-AS1 LOC100506157 ADAT2 CDKN1B BTRC EIF2B3 ACBD6 CEACAM22P GMCL1 FNIP1 SLC26A3 SLC2A3 COPG1 FAM102B KIAA1143 NUP153 HSD17B4 MRPL48 C9orf41 STIM1 CAMK2D KNSTRN HAUS6 INTS8 MYNN DHRS13 LPXN DSC2 PSMG2 PPIG SUN1 LDAH SMC4 MTF1 TM2D3 TMEM171 TMPO LYRM5 NR2C2AP RNF5P1 UBR5 NBPF1 STK38L C4orf19 MORC3 MRRF PRKAB2 CCDC32 RAF1 SGOL1 SMIM12 PLS1 CWF19L1 PDE8A ARPP19 GGPS1 C11orf85 PSMB2 SMIM7 BEST1 LOC100506844 COX20 DENND4B PPP2R5C ROBO1 MYLK4 ADAL CWC27 ITPR1 EBF3 EIF5A2 RAD51C IRAK3 DGKB TTC32 CDH26 SETD5 ABI3BP CTH LOC100128851 PCID2 KCNK1 TMEM55B DCAF7 RNFT2 PARP2 PMS2L2 ISG20L2 ZSWIM6 KIAA2026 ESD AACSP1 COQ3 RBL2 SRGN MRPL50 CIR1 STYX NDUFB6 GABPB1 PFKM NHEJ1 GART TRIAP1 ERCC3 PNPLA8 RFC4 DRG1 SRP19 DCAF16 RNF138 PBK HIST1H2AI AIMP2 COA1 EHBP1 SKA1 ZRANB2 FAM149B1 CCND3 DCTN6 MGARP CDC7 SEC31A PSMD14 KDM3B MRPL44 PSTPIP2 C1orf109 CASP3 ITGB3BP RNF213 CUL1 ZNF461 SIAH1 NCKAP1 CLK1 BTF3L4 TUBE1 SLC37A4 DDX19A ZNF45 TK1 C10orf88 NUDT2 BRD9 CCDC84 FAM103A1 PRKRIR NIP7 MED4 SS18 SHCBP1 PPARA KRIT1 CHM TVP23B HERPUD1 CCNL1 CDCA3 SEPP1 LAMB1 BRIP1 TRIM21 ETFDH TRIM13 CLECL1 DENND1B TNPO3 NELL1 MTURN HTT KDM4C NUP160 DDIT3 CASC5 C9orf84 FIGNL1 TDRD12 LOC100505543 COX18 GTF2IRD2 XPO1 ATG4C EPG5 TROVE2 IFI30 AUNIP CEP76 RRM2 MRPL2 COPS8 BFAR TOP2A PPIL2 MRPS23 IPO7 STAG3L1 LOC100507250 JARID2 RBM22 TUBG1 LOC100505991 SLC25A6 KRR1 SPC25 RABEP1 TRIM39 LOC283440 LARP1B KIF15 AP3S1 GSTM3 LOC100287290 NFATC2IP LNX2 ZEB2 RIT1 POLR2H NSA2 CCNH USP48 NR1H4 SAP30BP PPAT KIF24 NMI KIF13B SUGP2 ANKRD12 RBM18 ADAMTS13 SLC30A5 ZC3H11A LOC100505648 RNASEH2A C18orf25 GGNBP2 ATAT1 REL INSR ARHGEF6 LLPH WDR41 RBBP5 ILKAP HLA-B SCAND2P PDS5A CUL2 MORN4 PSMD13 IARS2 UBIAD1 FAM35BP NUTM2A-AS1 TMX2 ZNHIT3 TMEM126B FBXO48 TJP2 PEX19 PRKAB1 TM2D1 PLB1 PNO1 SPATA13 MARS CLIP1 CENPC1 SEMA5A MAK TTPAL ZNF222 F11R TMEM254 DTWD1 KDM5D SNRNP27 LOC100132815 DNAJC24 BCAT1 LOC100505795 LOC100506473 LRCH3 THAP6 ATG16L1 STRN PSMB10 GIT2 C21orf58 TDP2 CYP24A1 SCNM1 SUSD1 ATP8B1 CLIC6 CENPM GRIA2 PRKAG1 LOC643339 MDM4 PUM1 SMCR8 SELO HBA1 SLC39A6* |
| Unique gene names for CD71^++++^ (1288) | 657 | *MSRB1 SLMAP DPY30 TFIP11 PIGX GNL3 BTG2 UTP20 MTERF ANKRD36B PDCL3 U2SURP RPS18 POLK PAK1 TPX2 TECTA NEPRO TAF9B MLF1IP TRIM33 LOC93622 PNPT1 COX15 NSMCE2 ZNF582-AS1 DMC1 EPT1 DARS CCDC34 BMP3 SLK LUZP1 HNMT GRPEL1 BAZ1B SDAD1 PPP1R15B LARP7 SMC3 DCP1A PRDM2 TSPAN13 STAU2 EXOSC2 RBPJ EXOSC7 UFM1 FBXO11 SRSF6 GRB2 HIKESHI EP400 GCC2 WBP4 HNRNPR ENOSF1 CAMTA1 NPTN-IT1 ASUN SON RACGAP1 NDUFS4 UBXN4 PIK3CB ERBB2IP FUS TASP1 NIPBL DDX18 PPP2R5A DEPDC5 LTBP1 SENP7 APPBP2 ANKRD49 SUV39H2 PSMC3IP KDM6A SPG20 GLG1 MIS18BP1 KIF2A MNF1 CBFA2T2 C21orf59 WDR12 NOP58 FRYL ZNF432 SF3A3 RPP14 FAM175A CAMSAP1 RTCB RBM6 METTL21D MAGOHB GOT2 POLD2 MOGS KIN SSRP1 RPS17L CHAF1A MTMR6 CENPJ NPRL2 RAD1 C12orf65 NDUFAF8 OSER1 NCAPD3 CNDP2 LRRC42 MGC70870 ZMYM5 MIPEPP3 MEMO1 RPL21P28 ZNF518B RAD54B LRR1 MCFD2 EPS15 BDP1 WBP11 MCM10 ANKRD17 PRKDC FAM72D PSIP1 BCAS2 TAF1D KIAA0368 LOC100631377 CEBPZ MCM5 RABGGTB LOC101060276 FASTKD2 GEMIN6 SF3B2 LIMS1 PAK1IP1 SLC7A11 DERL2 CCDC14 UBE2L3 TXNDC12 SUPT16H POP4 CCNA2 ABT1 SMG1 PCYT1B GPD1L PSMA5 MAPK1IP1L CCDC28A PPP4R1 CNOT2 TWSG1 C8orf59 SNW1 RPP30 EBP LOC727820 TIMM8A SRRM1 TRIP11 SEPT7 USP38 ARHGDIB PHAX IFT74 FAM21A PNISR RWDD2B FH HDDC2 CCDC174 DIAPH3 CTR9 INTS7 GLO1 TRPM7 PPIL3 RBM34 EXOC4 CBWD1 LOC100129518 METTL14 ACADM CMSS1 C5orf63 NAMPT KIDINS220 FNIP2 ETV3 G3BP2 GTF2A2 ZC3HC1 SNHG15 TDRD3 BIVM BLVRA VPS4B ENTPD1-AS1 MED7 CDK13 SNX9 ANKRD36C GLMN AGAP6 GLT8D1 RIOK2 TRDMT1 SKIV2L2 ABCF1 FCF1 SMARCA4 CENPW TCEB1 BOD1L1 RTRAF SEC61G FBXL22 TCERG1 RTCA HDAC8 SRP54 RNASEH2B RFC1 MTAP RBBP7 SLC30A1 SNRNP25 MTFP1 RAD50 GUF1 TGS1 YTHDF2 CEP192 TARDBP RB1 KIAA1731 PWP1 DHX29 BZW2 METAP2 GLRX MRPL27 GPAM DDX10 RPA3 C12orf45 ZNF730 PTPLB TOMM70A ESCO2 TAF8 NUDT13 PPP6C PSMC5 ZCCHC11 C16orf87 BAG5 PPA2 PPIP5K2 TRAF3IP3 MGAT3 GTF2H2C TANGO6 EBNA1BP2 CUTC PTRH2 METTL13 NDUFA12 HOOK1 LAP3 PPM1G ELMSAN1 CHURC1 TBRG4 RAB1A RNMT MRPL42 CENPK AREL1 MRPL51 RBP3 TMEM167B PLEKHG1 MINOS1 ZNF789 SEPT11 ADSS MTIF2 DNAL1 IDE EIF3CL SFXN4 HIST1H1D POLR3GL WDYHV1 LPIN1 KCTD3 LIN7C CCP110 APC SNORA71C RSL1D1 RBM25 N6AMT2 ARCN1 MECR LOC441124 KIAA0101 TPR TTC4 CNOT11 NAT1 LOC100288152 BACH2 MRPL30 C8orf33 SCAF4 EPM2AIP1 RBM28 KTN1 HSBP1 RBAK SMIM11 RCN2 GINS2 LYPLAL1 LBR RAP1GDS1 CDC25A CCDC25 UGCG SUPV3L1 ORC3 ATRX RCBTB1 CCDC59 MNAT1 ANXA1 SLTM MATR3 CLGN HMGB3 HLTF TOPBP1 CTNNBL1 GNAI3 SPATA5L1 PTPLAD1 KDELC2 TRIM22 FKBP7 WASH1 PNPLA4 ACTL6A TXLNG2P CTDSPL2 ANKRD10 PHF3 ZNF148 TEKT4P2 CAPZA2 SFR1 TMEM222 MOSPD2 COMMD2 RPA2 MIER3 CHEK1 TRNAU1AP MRPL47 DYNLT1 APOL4 SPOPL SNORA40 LARS ALG14 TRMT10B MED8 WDR75 FAM216A DYNC1LI2 COIL ATP7A EMG1 PPP2R1B MARCH5 KIFC1 EVI5 NT5C ATP2C1 CSTF2T PARP14 CDR2 ZFP36L2 NAA16 VAPA SMIM8 CHCHD7 TRAPPC2L HDGFRP3 CCDC138 MCM3 C17orf75 SLC43A3 SS18L2 CDK6 TMTC3 QPCTL SUB1 MRPS33 NAA20 RHNO1 KIF20B MED11 CPSF3 TIMM10B SENP6 RIF1 SRBD1 WARS SNRPD1 MEPCE CCDC158 GPATCH4 ABCE1 REV3L TTC5 KYNU ZCCHC7 CCDC42B NOP16 ANKRD11 KIF2C VPS11 SPEN NDUFB5 LOC100506548 FBXO5 CHCHD1 PTER MRPL35 MPHOSPH8 CCBL2 ATM PRRC2C WDR60 THG1L TNFAIP8L1 SMEK1 RPL13AP5 CSE1L LCA5 RBM12B FAM118A CBWD5 SETD5-AS1 MRPL17 TADA2A PPT1 CCNT2 ELMOD2 DYNC1I2 TADA1 POLQ VPS13D ATL3 C3orf37 CTCF SRSF4 TROAP USP37 FBXO43 RPF2 MTPAP UBE4A FXR1 LSM7 NDUFC2 TRAPPC13 FAM60A ITGA4 PRPF40A KATNBL1 CEP57L1 CPNE8 PSMA4 ROCK1 TAGLN ZC3H13 DPH5 TMBIM4 GRB10 LANCL3 PRPF4 SACS LRP6 NT5C3A MRPL32 CAPZA1 TFB2M CPSF1 PYGL JKAMP NOD1 FAM136A HIBCH CYP20A1 LOC100505633 SNAP23 HSPA9 LOC100505964 SRSF10 CKLF ZNF451 RAB3D SRPK2 DHX57 ITPA CALU TDG FIP1L1 C4orf21 CKAP2 MARCH6 PABPC4 DIAPH1 AKAP9 AIMP1 SF3B1 HPRT1 ATRIP FAM69A RAD54L TNNI3K FAM172A SMU1 PRPF18 TMEM138 WDHD1 TMCO1 LOC100233156 QTRTD1 WNK3 TXNDC9 DDX54 GOLIM4 WRN ANAPC5 MRP63 JMJD1C BAZ1A NSRP1 ARID4A TRMT12 NVL FBXO4 DTL SRFBP1 CREBRF IFT81 TM7SF3 INTS4 CDC123 TTN HLA-DMA MTERFD2 CIT IER3IP1 ACN9 FLJ32255 RANGRF PTBP2 HMMR QSER1 NOLC1 RHOBTB3 ADIPOR2 ANKIB1 SART3 SNRPD3 TSR1 GMNN RSRC2 SPCS2 NNT UCHL5 TIMM21 POP5 PPIE GTF3C6 NUDT5 MORN2 TRAPPC2P1 ANAPC15 DIS3L BCL11A SAMD9 WASH3P CBX5 LRRC16A PPP2R5E GMPS SNRNP40 CDC5L TEX30 MAMDC2 COA3 GTF2H3 RANBP2 ATIC HIST1H4C PNN TXNDC17 MARVELD2 ZMYM6 TNFAIP8L2 NCAPG PCED1B-AS1 CCDC112 FTSJ2 DKFZP586I1420 GOLGA4 ATPAF2 SUCLA2 IDH3B DAAM1 SRF SMARCE1 DNAH5 VRK1 C2orf74 USP1 SMEK2 SAR1B RNF6 FANCB SNORD8 FAM49B ATP5G1 PPWD1 EAF2 GNL2 PRR3 SGPP1 CDCA8 H2AFY* |
